# Multiomics analysis of naturally efficacious lipid nanoparticle coronas reveals high-density lipoprotein is necessary for their function

**DOI:** 10.1038/s41467-023-39768-9

**Published:** 2023-07-06

**Authors:** Kai Liu, Ralf Nilsson, Elisa Lázaro-Ibáñez, Hanna Duàn, Tasso Miliotis, Marie Strimfors, Michael Lerche, Ana Rita Salgado Ribeiro, Johan Ulander, Daniel Lindén, Anna Salvati, Alan Sabirsh

**Affiliations:** 1 Advanced Drug Delivery, Pharmaceutical Sciences, R&D, AstraZeneca, Gothenburg, Sweden; 2 Translational Science and Experimental Medicine, Research and Early Development, Cardiovascular, Renal and Metabolism, BioPharmaceuticals R&D, AstraZeneca, Gothenburg, Sweden; 3 Bioscience Metabolism, Research and Early Development, Cardiovascular, Renal and Metabolism, BioPharmaceuticals R&D, AstraZeneca, Gothenburg, Sweden; 4 Data Science and Modelling, Pharmaceutical Sciences, R&D, AstraZeneca, Gothenburg, Sweden; 5 Division of Endocrinology, Department of Neuroscience and Physiology, Sahlgrenska Academy, University of Gothenburg, Gothenburg, Sweden; 6 Department of Nanomedicine & Drug Targeting, Groningen Research Institute of Pharmacy, University of Groningen, Groningen, 9713AV The Netherlands

**Keywords:** Nanoparticles, High-throughput screening, Drug delivery, Gene delivery

## Abstract

In terms of lipid nanoparticle (LNP) engineering, the relationship between particle composition, delivery efficacy, and the composition of the biocoronas that form around LNPs, is poorly understood. To explore this we analyze naturally efficacious biocorona compositions using an unbiased screening workflow. First, LNPs are complexed with plasma samples, from individual lean or obese male rats, and then functionally evaluated in vitro. Then, a fast, automated, and miniaturized method retrieves the LNPs with intact biocoronas, and multiomics analysis of the LNP-corona complexes reveals the particle corona content arising from each individual plasma sample. We find that the most efficacious LNP-corona complexes were enriched with high-density lipoprotein (HDL) and, compared to the commonly used corona-biomarker Apolipoprotein E, corona HDL content was a superior predictor of in-vivo activity. Using technically challenging and clinically relevant lipid nanoparticles, these methods reveal a previously unreported role for HDL as a source of ApoE and, form a framework for improving LNP therapeutic efficacy by controlling corona composition.

## Abstract (web summary)

ApoE is known to be important for lipid nanoparticle function. Here, the authors shows that efficacious coronal ApoE originates from high-density lipoprotein (HDL) particles and, enhances hepatic delivery, making HDL a superior biomarker for lipid nanoparticle potency.

## Introduction

Lipid nanoparticles (LNPs) are the lead non-viral delivery system for clinical nucleic acid therapeutics^1^. LNPs usually contain cationic ionizable lipids (CILs), helper phospholipids, cholesterol, and polyethylene glycol lipid (PEG-lipid)^2^. Microfluidic, rapid mixing techniques can formulate reproducible sub-100 nm LNP batches at a large scale^3^, allowing LNPs to progress quickly from concept to clinical applications^4,5^. The SARS-CoV-2 vaccine development has recently highlighted the utility of LNPs as mRNA delivery vehicles^6^. A one-size-fits-all LNP formation and mRNA dose has, however, been utilized to meet the urgent need of vaccinating the general public, regardless of age, gender, and individual physiological or pathological diversities.

So far, improvements to LNP engineering have been predominantly a result of the particle-centric factorial and combinatorial engineering that has optimized the nanoparticle components, composition and/or formulation^7^. Individual physiology and pathology are, however, confounding factors for LNP design, and the high-throughput tools necessary to derive the relevant engineering principles have not been available^8,9^.

Recently, modulation of biomolecular corona formation (and thus content) has become a promising strategy for improving LNP potency^10,11^ and extrahepatic targeting tropism^12^, linking physiology to design. The biomolecular corona, formed on LNPs following administration, contains hundreds of biomolecules (primarily proteins and lipids) that create a new biological identity for nanoparticles^13^. Corona composition is highly variable and is strongly influenced by individual pathophysiology, underlying disease, and co-medication, affecting nanomedicine targeting, clearance, distribution, and cargo release^8^. For example, the biodistribution of nanoparticles in obese animals deviates significantly from their healthy counterparts^14^. Therefore, deciphering corona composition on an individual basis will offer more insight than standardized conditions and contribute mechanistic knowledge regarding corona modulation of LNP potency.

Among all LNP corona biomolecules, Apolipoprotein E (ApoE) has been most intensively studied, notably as a targeting ligand to improve LNP delivery to the liver in clinical formulations^15,16^. Interestingly, ApoE (Apoe−/−) and LDLR (Ldlr−/−) knockout mice did, however, indicate the LNP potency was only dependent on the presence of ApoE, not LDLr^17^, suggesting more complex machinery involving ApoE. Furthermore, while most mechanistic studies have focused primarily on unbound, free ApoE, a large proportion of ApoE in blood plasma associates with various endogenous lipoprotein particles, creating a greater diversity of biomolecules and higher complexity^18^. ApoE can also actively incorporate into LNPs, affecting both their structure and composition^19^. These examples highlight the need to mechanistically understand corona-centric LNP potency modulation, ideally in various physiological or pathological settings with unbiased high-throughput methodologies more suited to factorial and combinatorial LNP engineering.

The technical challenges associated with isolating LNPs and their associated corona components are a major obstacle. Compared to other nanoparticles, current methods for LNP corona isolation are generally laborious, low-throughput, consume relatively large amounts of expensive materials, and often require additional particle modifications or methods that can perturb the corona composition and integrity^20^. Most unfavorably, they cannot guarantee a distinct separation of LNPs from vastly more numerous and physically similar blood plasma nanoparticles such as lipoproteins or extracellular vesicles^20–23^. In addition to proteins, lipid species and nucleic acids were found at the surface of nanoparticles identified by multiomics analysis in other lipid-based nanoparticles^24,25^. They also likely influence the efficacy and pharmacokinetics of nanoparticle-based therapeutics in vivo^26,27^. Therefore, there is also a need for technically demanding multi-omics phenotyping of the biomolecular corona surrounding LNPs.

This study explores the impact of individual physiology on corona formation using an obesity model to reveal corona components that drive differences in LNP efficacy. Using various plasma dilutions to reveal the underlying pharmacology, a clinically mature LNP formulation was functionally evaluated in-vitro, following incubation with plasma obtained from individual lean or obese rats. An ultrafast, affinity-based, automated high-throughput corona isolation method, coupled with downstream omics analysis, was used to quantify the corona composition of the same LNP-corona complexes used in the functional experiments. The protein and lipid corona, analyzed by unbiased quantitative multi-omics, revealed the correlation between the corona components (high-density lipoprotein (HDL) in particular) and LNP efficacy. The resulting mechanistic insights were then validated in-vivo. Our results improve our understanding of how corona components affect LNP efficacy, with implications for future mRNA vaccine development, personalized nanomedicine design, and human clinical translation.

## Results

### Lean and obese animals, ApoE and the effects on LNP potency

**Fig. 1:**
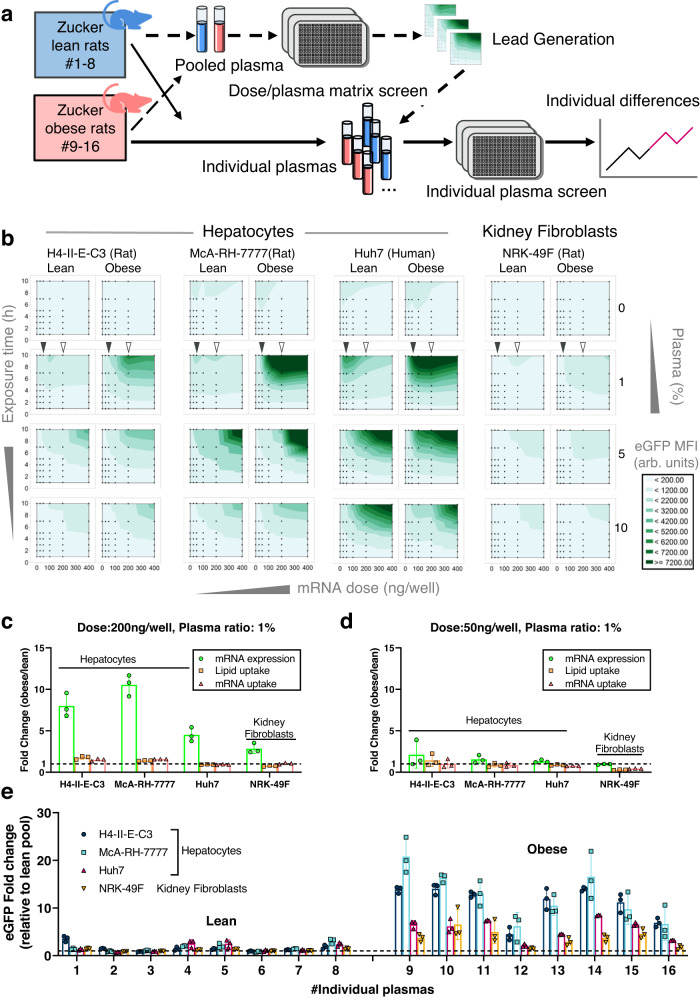
The efficacy of LNPs mRNA delivery is individual plasma and dose dependent. **a** The experimental design for evaluating LNP potency under lean and obese conditions. Lean pool (LP) and obese pool (OP) plasmas from individual samples were used for initial lead generation to evaluate LNP functional dose-response relationships and identify key corona components, followed by an evaluation of selected LNP doses in combination with individual plasma samples. The candidate mRNA doses and the LP and OP plasma concentrations resulting in the largest difference between lean and obese states were identified prior to exploring individual plasma samples. **b** A series of mRNA doses (25–400 ng/well) were tested in the presence of LP or OP plasma (0, 1, 5. and 10%) using H4-II-E-C3, McA-RH-7777, Huh7. and NRK-49F cell lines over a 10 h time course. The cellular eGFP mean fluorescent intensity (MFI) was quantified using image analysis and visualized as contour maps (each black dot represents the mean of *n* = 3 experimental replicates). In general, the eGFP expression increased over time. The obese plasma induced higher eGFP expression at the 1% plasma concentration in hepatocytes. Source data are provided as a Source Data file. **c**, **d** The fold change of mRNA cargo expression (eGFP) and LNP uptake (Rhodamine label for lipids; and Cy5 label for mRNA) at the 10 h endpoint was calculated using the data from panel b as indicated by triangles (solid triangle: 50 ng/well dose; hollow triangle: 200 ng/well dose). At the 50 ng/well mRNA dose, the lean and obese plasma-complexed LNPs resulted in comparable cellular uptake and eGFP expression. The uptake of LNPs, following lean and obese plasma supplementation, was mildly improved at the 200 ng mRNA/well, whereas the eGFP expression was significantly elevated, particularly in rat hepatocytes. The values are derived from raw images (*n* = 3 experimental replicates). Source data are provided as a Source Data file. **e** The difference in LNP efficacy was assessed using a 200 ng/well mRNA dose in vitro. An apparent variation was observed between lean and obese plasmas, and among every individual. The error bars represent standard deviation of the mean values derived from raw images (*n* = 3 experimental replicates). Source data are provided as a Source Data file.

To begin our search for naturally efficacious LNP coronas and to investigate how pathological variation of individual plasma content might influence LNP-mediated mRNA expression, we compared eGFP mRNA expression in-vitro using cell cultures supplemented with plasma withdrawn from obese and lean Zucker rats (Crl:ZUC-Lepr^fa^). The obese Zucker rats exhibit spontaneous hyperlipoproteinemia and a lower level of hepatic low-density lipoprotein receptor (LDLr), resulting in an elevated number of very-low-density lipoprotein (VLDL) and HDL particles in plasma^28^ (Supplementary Fig. 1). A range of obesity biomarkers were significantly elevated in the obese samples: cholesterol, triglycerides, natural phospholipids (Supplementary Data. 1), and lipid binding apolipoproteins, including lipid binding ApoE (Supplementary Fig. 2). Figure 1a illustrates the screening procedure to evaluate the effects of the individual plasmas on LNP mRNA delivery using both individual plasmas and pooled lean (LP)/obese (OP) plasmas from individuals.

The LNPs were formulated using the clinically approved cationic ionizable lipid DLin-MC3-DMA (MC3) and eGFP-encoding mRNA, with a small portion of the helper lipid and mRNA cargo substituted with their respective rhodamine (Rhod) and cyanine 5 (Cy5) labeled counterparts (Supplementary Fig. 3a). The LNPs, characterized using Dynamic Light Scattering (DLS) and Electron Microscopy (EM), had a diameter of ~80 nm and a spherical morphology with an electron-dense core (Supplementary Fig. 3b, 3c, and Supplementary Table 1).

We then created a factorial in-vitro model system that uses various plasma concentrations to create different ratios between the number of LNPs dosed and the endogenous plasma components, simulating a variety of physiological conditions and unmasking the three-way pharmacological interaction between the LNPs, the plasma components, and the cells in a way that is not feasible using in-vivo experiments. High-throughput confocal live-cell imaging was employed for quantitative screening of cellular LNP uptake and productive cargo delivery, using human and rat hepatocytes or rat kidney fibroblasts (as a non-liver alternative) and either plasma-free media or growth media supplemented with 1–10% LP or OP plasma. The cells were then treated with LNPs using a dose range from 25 ng to 400 ng mRNA/per well (0.5–8 µg/mL), and imaged at intervals over the next 10 h (Supplementary Figs. 4 and 5).

Analysis of the imaging data revealed that under certain conditions, obese plasma could improve the efficacy of LNPs. As shown in Fig. 1b, biomolecules and corona formation are indispensable for LNP-mediated uptake and mRNA expression. There was minimal LNP cellular uptake and negligible eGFP expression across all tested mRNA doses in the hepatocyte cell lines without plasma. Comparing LP or OP plasma-supplemented samples, the most noticeable difference in eGFP expression was when recipient cells were dosed within 1% plasma. Specifically, the most significant difference appeared when 1% plasma was combined with a 200 ng mRNA/well LNP dose. LNP efficacy was 5 to 10-fold improved by obese plasma supplementation at the endpoint (Fig. 1c and Supplementary Fig. 6). At lower LNP doses, the contrast between lean and obese plasma effects on LNP efficacy was less apparent (Fig. 1d). Meanwhile, the expression difference between obese and lean was less pronounced in kidney fibroblasts than in hepatocytes.

This model also revealed that higher protein production is not simply because of a general anabolic effect caused by obese plasma. For example, the higher ApoE content (i.e., obese vs. lean, or 10% vs. 5% plasma) did not significantly increase eGFP expression levels in recipient hepatocytes within the dose range evaluated. In contrast, 1% OP supplementation resulted in the highest eGFP expression, suggesting a more specific mechanism than anabolic upregulation of protein production. The cellular response pattern (Fig. 1b) also implied that the difference in eGFP expression did not directly correlate to the overall nutrient content of the plasma.

To further explore how individual physiological variations can affect LNP efficacy, we dosed cells with 50 ng and 200 ng eGFP mRNA containing LNPs, supplemented with individual plasmas at the same 1% ratio, and found the difference between lean and obese conditions remained. Individual variation was observed (Fig. 1e and Supplementary Fig. 7), although the intragroup variation (among lean or obese individuals) was less than the intergroup variation (lean vs. obese).

Based on these findings, we hypothesized that there should be a detectable difference in corona content between the obese plasma-LNP ratios that resulted in the most effective cargo delivery and, the corresponding lean plasma-LNP complexes. In addition, there might be an optimal ratio between the plasma components, the LNPs, and the cell surface. In the 1% lean plasma condition, lowering the plasma ratio may also unmask corona interactions with recipient cells, as previously reported^29^ and in this case, the limited availability of biomolecules necessary to form the biomolecular corona, especially at LNP doses higher than 50 ng mRNA/well, led to the attenuation in the eGFP expression. In contrast, the obese plasma facilitated eGFP expression under the same conditions and enhanced the expression at higher LNP doses, suggesting that the resulting corona composition was more compatible with hepatocellular delivery.

### Ultrafast affinity isolation enables unbiased biomolecular corona phenotyping

To derive the mechanistic understanding of the observed functional differences, and create principles for engineering desirable coronas, it was necessary to quantify LNP-corona components. This required separating LNPs, as intact particles with intact coronas (LNPcor), from unbound biomolecules and physically similar endogenous nanosized structures in the plasma (Supplementary Fig. 8). Commonly used methods to retrieve the LNPcor from plasma are typically slow and/or arduous, and not compatible with high-throughput screening. Recent studies have shown that size exclusion chromatography (SEC) methods are prone to co-isolate unbound protein and endogenous particles^20,21,30^. We assessed asymmetric flow field-flow fractionation (AF4), an improved size-based separation technique that has previously been utilized to isolate other lipid-based nanoparticles^31^. In agreement with the literature, isolation approaches for LNPs utilizing size or density fractionation were insufficient for retrieving LNPs from plasma because of the unfavorable overlap with other plasma components (Supplementary Fig. 9). This problem is unique to clinical lipid nanoparticles and differentiates them from other nanoparticles that are easier to separate analytically from biofluids.

**Fig. 2:**
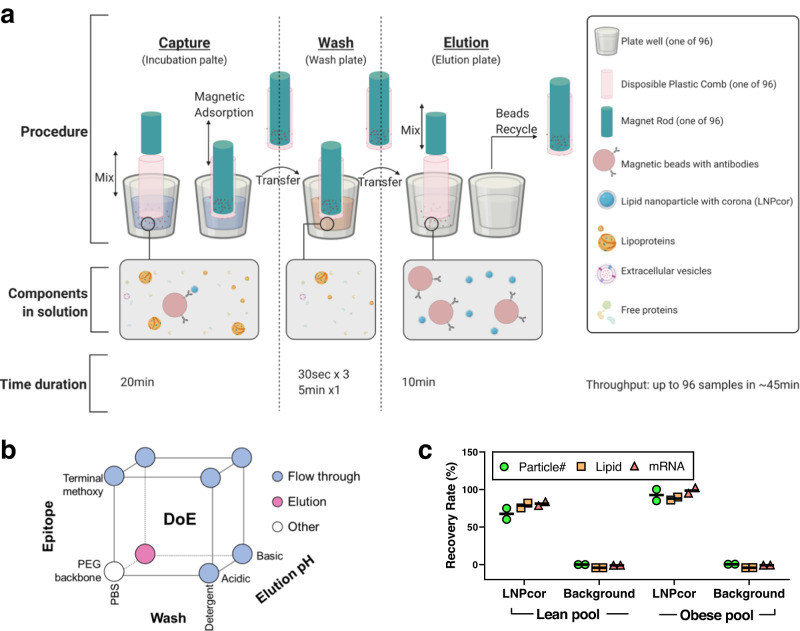
The development of affinity-based magnetic isolation of LNPcor complexes. **a** Schematic illustration of the ultrafast affinity-based, 96-well isolation method. Anti-PEG antibody-conjugated magnetic beads capture LNPcor from plasma containing free protein, extracellular vesicles and lipoprotein particles. Non-specifically bound biomolecules are removed with a series of wash steps. The LNPcor is eluted by alternating the pH. Up to 96 samples can be harvested in parallel within ~45 min. Illustration is generated with BioRender. **b** The design of experiment (DoE) space of LNPcor capture (epitope), wash and elution. As one of the LNPs’ structural components, PEG existing on the LNPs surface was employed here as a primary “endogenous” affinity tag, avoiding the need to add any additional surface antigens that might modify the LNPs’ surface properties and affect the biomolecular corona formation. To preserve the corona content and the LNPs themselves it was necessary to avoid commonly used detergents during washing and elution steps. A moderate change of pH was an effective and relatively mild way to disassociate LNPcor from antibodies. Nevertheless, a pH lower than the pKa of cationic ionizable lipid included in the LNPs (i.e., MC3, pKa = 6.44) usually results in LNP disassembly. The circles indicate where the majority of LNPs were detected. Only when combining an antibody against PEG backbone with PBS washing and basic elution conditions, were the majority of LNPs was identified in final elution. **c** The recovery ratio of LNPcor in terms of particle number, rhodamine labeled lipids (lipid) and Cy5 labeled mRNA (mRNA). Mean value (black bar) derived from independent experimental replicates (*n* = 2), with individual values indicated. Source data are provided as a Source Data file.

To avoid these problems, we developed a fit-for-purpose affinity-based, magnetic isolation workflow (Fig. 2a) employing antibodies against the endogenous polyethylene glycol (PEG) on the surface of LNPs, using a miniaturized, high-throughput 96-well format. The isolation procedure contains three essential steps: LNPcor capture, wash, and elution. A statistical design of experiments (DoE) method was used to optimize these three steps (Fig. 2b). A combination of antibodies against PEG backbone, PBS wash and basic pH elution resulted in robust and specific LNPcor recovery in the final elution. Optimal incubation and elution conditions that resulted in maximal LNP retrieval without impurities were also identified (Supplementary Fig. 10).

As shown in Fig. 2c, this method retrieved the majority of the LNPcor complexes, with a similar recovery profile for LNPcor formed in both lean and obese plasma samples. The number and recovery rate of nanosized particles within the elution was examined by Nanoparticle Tracking Analysis (NTA) to confirm that LNPcor harvested particle-like material. The recovery rate of lipid and mRNA content was quantified using fluorescence intensity. Overall, the recovery rate of nanosized particles matched lipid and mRNA recovery, indicating very little lipoprotein contamination. Controls using antibody-conjugated microbeads incubated with 1% LP or OP in absence of LNPs confirmed negligible unspecific binding of endogenous nanosized particles. This automated affinity-based magnetic isolation workflow had superior specificity compared to conventional methods and, the entire isolation procedure for up to 96 samples can be completed within ~45 minutes, minimizing corona perturbation while maintaining throughput.

To verify the integrity and functionality of the LNPs following the isolation procedure we retrieved the LNPs from PBS (LNP pull-down or LNP_PD_), lean pool (Lean_PD_), or obese pool (Obese_PD_) plasmas following a 4 h incubation at 37 °C. Compared to processed LNPs, the mRNA encapsulation was not reduced during the isolation or incubation procedures (Supplementary Fig. 11a). The original LNPs and LNP_PD_ were also evaluated functionally. The two types of LNPs provoked similar eGFP expression in recipient cells, and the responses to lean or obese plasma was consistent with previous results, indicating that LNP functionality remained unperturbed (Supplementary Fig. 11b). There was also negligible cellular uptake of the original LNPs in plasma-free conditions. In contrast, LNPs with fully formed biomolecular corona were taken up by cells even in the absence of plasma, demonstrating that the isolation procedure can harvest LNPcor with functional coronas (Supplementary Fig. 11c).

Cryogenic transmission electron microscopy (Cryo-EM) was also employed to reveal the macroscopic effects that the corona exerts on LNPs (Supplementary Fig. 12). In line with earlier descriptions, the LNPcor formed in LP plasma appeared mostly as electron-dense spheres^2^. Intriguingly, the OP LNPcor had multi-laminar structures, likely resulting from incorporating biomolecules with detergent-like properties, such as apolipoproteins^32^. DLS revealed that the obese-plasma-derived LNPcor had, on average, a significantly larger hydrodynamic diameter than the lean-plasma-derived particles, while the polydispersity was lower (Supplementary Fig. 13a and 13b.).

Overall, the results confirmed that ultrafast affinity isolation can harvest functionally intact LNPcor while minimizing the impurities that biased conventional isolation methods.

### The physiological state of individuals alters LNP biomolecular corona formation and content

**Fig. 3:**
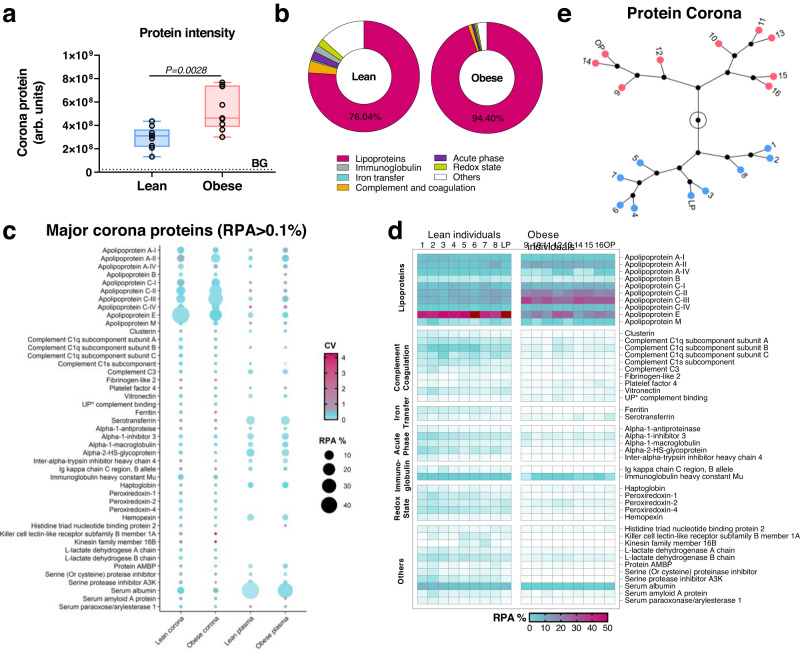
Proteomics profiling of individual biomolecular coronas. **a** The comparison between lean/obese plasmas derived LNPcor entities. The mean intensity of each group was summarized from all identified corona proteins from each independent individual-plasma- (*n* = 8) and the pooled lean/obese plasma (*n* = 1) derived LNPcor. The uncomplexed LNPs and non-specifically bound lipid binding were evaluated, respectively. For box and whisker plots, each data point represents an individual LNPcor, averaged from duplicate measurements. The box represents the interquartile range (first quartile, median and third quartile). BG is the intensity summarized from background impurity controls. *P* value is obtained by two-tailed Mann-Whitney test. Source data are provided as a Source Data file. **b** The average ratio of major proteomic functional clusters summarized from lean and obese-individual-derived LNPcor. Source data are provided as a Source Data file. **c** The most abundant coronal proteins (RPA > 0.1%) in lean and plasma-derived LNPcor and the corresponding RPA in original plasmas (*n* = 2). RPA: relative protein abundance. CV: coefficient of variation. Source data are provided as a Source Data file. **d** An overview of major corona proteins in individual LNPcor (*n* = 2). UP, uncharacterized protein. **e** Hierarchical clustering of individual plasma derived LNPcor using proteomic fingerprints. Lean- and obese-plasma-derived LNPcor formed distinct clusters. Source data are provided as a Source Data file.

We had tested LNP function following complexing with plasma from individual animals and the next step was to explore this mechanistically by linking LNP corona content to LNP efficacy at the individual level. Following method development with pooled plasma samples, we focused on characterizing LNPcor formed in individual lean and obese plasmas, and the individual coronas were evaluated using quantitative proteomics, revealing significant differences between lean and obese LNPcor. On average, obese-plasma-derived biomolecular coronas contained 1.8-fold more protein than those derived from lean plasmas (Fig. 3a). The unspecifically bound proteins (Fig. 3a, BG) and endogenous plasma particles were substantially lower than the LNPcor samples. The size and particle number of LNP, LNPcor, and relative background control were also visualized and measured by NTA. During each NTA measurement, a movie of particular events in Brownian motion was recorded. Representative movies demonstrating specific recovery of LNP and LNPcor are displayed as original LNPs (Supplementary Movie 1), PBS (buffer for sample dilution, Supplementary Movie 2), Lean LNPcor (Supplementary Movie 3), lean background (non-specific pulldown from lean plasma-beads interaction, Supplementary Movie 4), obese LNPcor (Supplementary Movie 5), obese background (non-specific pulldown from obese plasma-beads interaction, Supplementary Movie 6). The average relative protein abundance (RPA) of corona apolipoproteins increased from 76.04 to 94.40% when moving from lean to obese plasmas (Fig. 3b). The increase in lipid-binding, amphipathic, and apolipoproteins also suggests a potential explanation for the changes in the cryo-EM morphology of LNPcor formed in obese plasmas. Other consistently detected corona proteins were involved in the acute-phase response, redox homeostasis, iron transfer, complement and coagulation processes, and/or other immune responses.

In general, the LNP protein corona did not simply mimic plasma protein composition, implying selective absorption of corona proteins. Figure 3c illustrates the major corona proteins detected (RPA > 0.1%, summarized from individual coronas) and their abundance in the original blood plasma. We found that in obese-plasma-derived coronas, apolipoprotein A-II (ApoA-II), apolipoprotein C-II (ApoC-II), apolipoprotein C-III (ApoC-III), and apolipoprotein M (ApoM) were elevated. In contrast, the ApoE abundance was moderately reduced in all obese-plasma-derived coronas compared to corona samples derived from lean plasmas, despite the improved eGFP expression following LNP exposure to obese plasma (Fig. 1b). Immunoblotting was employed to confirm the presence of ApoA-II, C-II, C-III, and E in LNPcor (Supplementary Fig. 15). Figure 3d illustrates the major corona proteins detected in individual coronas. In Fig. 3e, individual protein corona fingerprints were evaluated using hierarchical clustering. Obese and lean-derived coronas self-organized into two clear and distinct groups, highlighting that while there is variation in corona protein content introduced by individual plasma samples, the lean and obese corona phenotypes are very distinct.

**Fig. 4:**
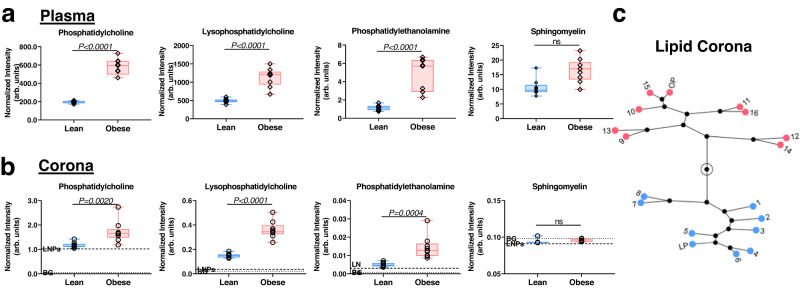
The lipidomics profile of corona lipids. **a** The summary of natural lipid species intensity identified within individual plasm. The mean intensity of each group was summarized from identified corona lipids from each independent individual-plasmas (*n* = 8) and the pooled lean/obese plasma (*n* = 1). For box and whisker plots, each data point represents an individual LNPcor averaged from duplicate measurements. The box represents the interquartile range (first quartile, median and third quartile). *P* value is obtained by two-tailed Mann–Whitney test. Source data are provided as a Source Data file. **b** The summary of natural lipid species intensity identified within individual-plasma-derived LNPcor. The mean intensity of each group was summarized from identified corona lipids from each independent individual-plasma-derived (*n* = 8) and the pooled lean/obese plasma-derived LNPcor (*n* = 1). Uncomplexed LNPs (LNP, dotted lines) and non-specific lipid binding (BG, dotted lines) were also evaluated. For box and whisker plots, each data point represents an individual LNPcor averaged from duplicate measurements. The box represents the interquartile range (first quartile, median and third quartile) with whiskers indicating minima to maxima. *P* value is obtained by two-tailed Mann–Whitney test. Source data are provided as a Source Data file. **c** Hierarchical clustering of individual plasma-derived LNPcor using lipidomics fingerprints. Lean and obese plasmas derived LNPcor formed distinct clusters.

Lipids are also usually bound to apolipoproteins, forming lipoprotein particles, and as demonstrated above, apolipoproteins are enriched in LNPcor. Therefore, we examined the content of naturally occurring lipids in the LNPcor as previously described^33^, revealing elevated lipid content in obese, plasma-derived coronas (Supplementary data 2). Four lipid families, phosphatidylcholine (PC), lysophosphatidylcholine (LPC), phosphatidylethanolamine (PE), and sphingomyelin (SM), were measured for individual plasmas and coronas, and the results are summarized in Fig. 4. While comparing the obese plasmas to the lean counterparts, we observed a universal elevation of all examined lipid species except SM. As with the proteins above, the individual lipid corona fingerprints were used for hierarchical clustering analysis, and obese and lean-plasma-derived coronas formed two distinct clusters, demonstrating again the prominent differences in corona content driven by obesity.

### Corona HDL, rather than ApoE, drives hepatic LNP mRNA delivery

**Fig. 5:**
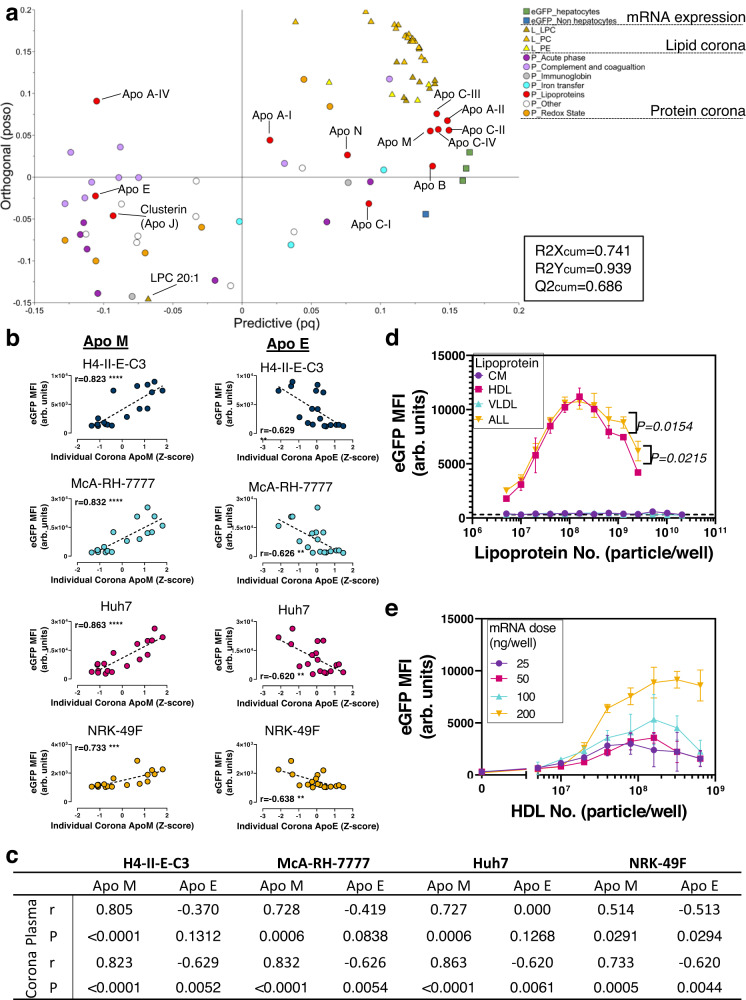
High-density lipoprotein modulated LNPs performance. **a** OPLS analysis to illustrate the correlation between corona contents and cellular eGFP expression. The orthogonal axis (orthogonal loading vector po of the X-part and the projection onto Y (so), poso) indicates the corona contents’ orthogonality to eGFP expression; The predictive axis (X loading weight p and Y loading weight q combined to one vector, pq) implies the corona content’s impact on eGFP expression. The distance on the *Y*-axis indicates orthogonality to the OPLS model. Omics hits that localize closer to 0 on the *y*-axis have lower orthogonality and are more relevant. While the distance along the *x*-axis reveals the relative correlation. **b** The correlation between corona ApoM or ApoE (z-score normalized) and eGFP expression in a variety of cell lines with Pearson correlation coefficient r with significance *p* value derived from *F*-test. ***P < 0.001, ****P < 0.0001. Exact *p* values are provided in **5c**. Source data are provided as a Source Data file. **c** The correlation coefficient r between corona and plasma ApoM or ApoE (z-score normalized) and eGFP expression in a variety of cell lines. **d** The spike-in of HDL, but not VLDL and CM stimulated LNP-mediated eGFP expression at 200 ng/well mRNA dose in Huh7 hepatocytes. Dotted line, the eGFP cellular expression level when supplemented with LP plasma. The error bars represent standard deviation of the mean values derived from raw images (*n* = 3 experimental replicates). Significance *P* values are determined by unpaired two-tailed *t*-test. Source data are provided as a Source Data file. **e** HDL spike-in with different doses of LNPs and HDL particles in Huh7 hepatocytes. The curves reveal the relationship between LNP and HDL particle numbers. The error bars represent standard deviation of the mean values derived from raw images (*n* = 3 experimental replicates). Source data are provided as a Source Data file.

The final step in our workflow was to develop and validate computational models for identifying LNP corona components that modulate LNP efficacy. Multivariate regression models usually outperform single-component models when using multi-omics data to predict the effects of corona composition on nanoparticle performance^34^. Therefore, we utilized an orthogonal partial least squares (OPLS) analysis to reveal the latent multivariant correlation between the individual plasma-derived biomolecular coronas and eGFP expression (Supplementary data 3). In Fig. 5a, the correlation between corona content (protein and lipid contents, X variables) and the eGFP expression obtained by quantitative imaging (AUC_10h_, Y variables) was illustrated as cumulative R2X, R2Y, and Q2 (R2X_cum_, R2Y_cum_, Q2_cum_, range 0–1). The correlation model explained 74.1% (R2X_cum_ = 0.741) of the X variable behavior and 93.9% (R2Y_cum_ = 0.939) of the Y variable behavior. An R2 value > 0.66 is considered indicative of good model fitness^35^. Furthermore, the overall model predictive power (X variables’ impact on Y variables) of the OPLS is 68.6% (Q2_cum_ = 68.6). The OPLS correlation model can therefore reveal the potential correlation between LNP performance and the corona content resulting from lean and obese plasmas exposure.

The OPLS analysis revealed two groups of corona proteins that were of particular interest concerning LNP efficacy: immune response proteins (exemplified by complement proteins) and, proteins related to lipid physiology (apolipoproteins). The immune response proteins are not considered here and are the focus of separate, ongoing experiments. The presence of many apolipoproteins in LNP coronas correlated with LNP efficacy (−0.1<Orthogonality<0.1). In particular, ApoA-II, B, C-II, and C-IV, the structural proteins found in HDL, VLDL, and chylomicron (CM) lipoproteins, were positively correlated with LNPs performance. ApoM, which is found primarily on HDL^36^, also exhibited a high positive correlation to LNP performance. In contrast, ApoE and ApoA-IV^37^, primarily found in VLDL and CM, had a moderately negative correlation to LNPs performance. While ApoE is often considered highly correlative to LNP hepatic delivery efficacy, the amount of corona ApoE in obese-plasma-derived coronas declined modestly compared to lean-derived counterparts (Supplementary Fig. 14). As shown earlier, when LNPs were complexed with obese plasmas, resulting in coronas with less ApoE, fluorescence imaging revealed more cellular eGFP mRNA expression (Fig. 1e).

While not all protein components of LNP coronas improve LNP function, most of the lipids associated with LNPs did correlate with enhanced LNP performance. A lipid-rich corona, in general, is favorable for LNP-mediated mRNA delivery. However, the orthogonality of lipid hits was higher than most of the protein hits (Orthogonality > 0.1 or <−0.1), suggesting that the corona proteins are more relevant for explaining the differences between LNP function in lean and obese contexts.

OPLS also revealed the intragroup correlation within X and Y variables separately. Within the Y variables group (eGFP expression), the human and rat hepatocytes were co-localized, indicating that the LNP performance with the three hepatocyte lines was similar when supplemented with either lean or obese plasmas, while non-hepatocytes were further away, demonstrating that LNP efficacy and the effect of corona content is dependent on cell type and tissue origin. Within the X variables, the apolipoprotein clusters (ApoA-II, C-II, C-III, C-IV, and ApoM) are adjacent to the corona lipids, suggesting that the apolipoproteins and lipids, which are commonly co-assembled into lipoprotein particles, formed LNP coronas collectively. In addition, the OPLS analysis revealed that acute phase, complement cascade, and coagulation function-associated proteins are less likely to be present in an apolipoprotein-abundant (and more functional) LNP corona.

Our focus then shifted to identifying the mechanisms driving corona formation and the associated efficacy variation in lean and obese conditions. While OPLS analysis revealed complex multivariant correlations and identified interesting components, linear classifiers are a more intuitive way to visualize the impact of corona components on LNP function. In Fig. 5b, c, we found a moderate correlation between individual corona ApoE and eGFP expression, but neither corona nor plasma AopE levels were optimal for predicting hepatic mRNA expression. In contrast, the correlation between individual ApoM levels and cellular eGFP expression was more robust. The corona ApoM also correlated more with LNP function than plasma ApoM. Other major HDL-associated apolipoproteins, such as ApoA-II, were also highly correlated with cellular eGFP expression (Supplementary Fig. 16).

We hypothesized that LNP corona formation involved interactions with various lipoprotein particles as intact lipoproteins or fragments have been identified in coronas^38,39^. It is challenging to map a proteomic fingerprint onto a naturally occurring lipoprotein particle population unambiguously due to the dynamics of lipid metabolism. Our data suggested that HDL particles were, however, the best match and a likely source of the efficacious corona components.

To explore LNP interactions with lipoprotein particles experimentally, Huh7 hepatocytes were exposed to LNPs together with 1% lean plasma spiked with purified HDL, VLDL, and CM to determine how these components affected cellular mRNA expression (LNPs supplemented with 1% lean plasma are less functional, Fig. 1). The lipoprotein candidates were spiked into lean plasma using a wide concentration range, either separately or combined with the LNPs prior to dosing, and the resulting eGFP production was measured (Fig. 5d, and supplementary Fig. 17).

Unlike other lipoproteins, only coronas augmented with HDL could potentiate cellular responses to LNP exposure. Even at the lowest spike-in concentration (5 × 10^6^ particles/well), HDL alone could potentiate cellular responses to LNPs, with a maximum response at 1.6 × 10^8^ particle/well. At higher HDL concentrations, a decline in eGFP mRNA expression was observed. In comparison, the addition of VLDL and CM did not, at any concentration, affect LNP performance compared to lean plasma alone (dotted line, Fig. 5d). When HDL, VLDL, and CM were spiked in an equal concentration together, the LNP performance enhancement was not different from HDL alone. In contrast, at higher spike-in doses, combining all lipoprotein particles mitigated somewhat the eGFP expression reduction seen when using higher doses of HDL.

**Fig. 6:**
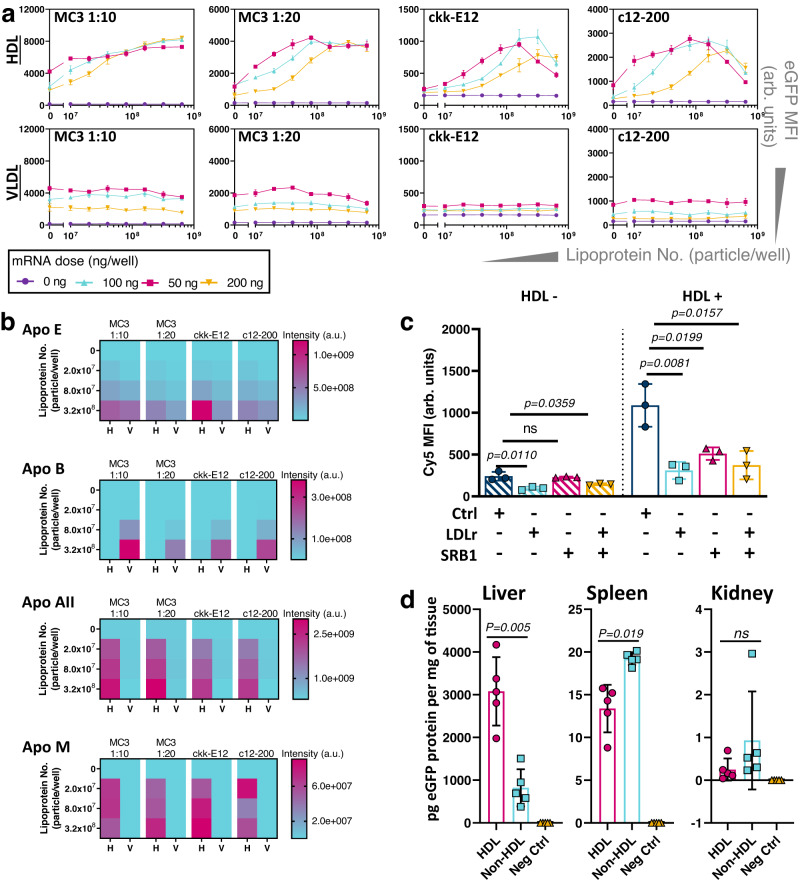
High-density lipoprotein functioned as a potent LNP efficacy modulator. **a** The spike-in of HDL, but not VLDL modulated LNP-mediated eGFP expression at 50, 100, and 200 ng/well mRNA dose with LNPs formulated with MC3 (lipid:mRNA = 1:10 or 1:20), cKK-E12 or c12-200 CILs in Huh7 hepatocytes. The error bars represent standard deviation of the mean values derived from raw images (*n* = 3 experimental replicates). Source data are provided as a Source Data file. **b** The intensity of corona apolipoprotein E, B, AII and M with various amounts of spike-in HDL(H) or VLDL (V). Source data are provided as a Source Data file. **c** The expression of the HDL receptors LDLr and SRB1 in Huh7 cells were inhibited separately or simultaneously with siRNA. LNPs were dosed to cells supplemented with LPDS. In the absence of HDL, inhibition of LDLr, but not SRB1, significantly reduced LNP uptake. With spiked HDL, the inhibition of both LDLr and SRB1 lessened LNP uptake. The type of siRNA treatments are indicated as +, treated and −, untreated. A scrambled sequence with no cellular target was used as the siRNA negative control (ctrl). The error bars represent standard deviation of the mean values derived from raw images (*n* = 3 experimental replicates). Significance *P* values are determined by unpaired two-tailed *t*-test. Source data are provided as a Source Data file. **d** Lean Zucker rats were dosed with eGFP mRNA containing LNPs or PolyA mRNA containing LNPs (Neg Ctrl, *n* = 5 independent rat) at a dose of 0.1 mg mRNA/kg body weight. The eGFP LNPs were preincubated in 1% LPDS with additional HDL (HDL group, *n* = 5 independent rat), or without HDL (Non-HDL, *n* = 5 independent rat) for 4 h to allow corona formation. The ratio of additional HDL to LNPs used was equivalent to the 8.0 × 10^7^ HDL particles/well condition used in (**a**, **b**). 6 h post administration, the rats were sacrificed and the expression of eGFP in liver, spleen and kidney was quantified using ELISA. With the addition of HDL, LNPs were more exclusively delivered to liver, with approximately four-fold higher GFP expression in liver and decreased expression in spleen and kidney. The eGFP signal in control group was below the detection limit. The error bars represent standard deviation of the mean values derived individual rats. Significance *P* values are determined by unpaired two-tailed *t*-test. Source data are provided as a Source Data file.

Next, we investigated how the HDL-potentiated cellular mRNA expression is related to the ratio between LNP and HDL. At lower LNP doses, the maximum mRNA expression occurred at lower HDL concentrations, while high LNP doses resulted in higher mRNA expression but required more HDL to reach the maximum levels (Fig. 5e), suggesting that there is an apparently constant optimal ratio between corona HDL and the LNPs in terms of productive cargo delivery. The decline of cellular eGFP expression at high HDL doses is likely a result of increased competition, between corona components and the unbound counterparts in plasma, for cell surface receptors (Supplementary Fig. 18). To further explore this, we retrieved LNPs with coronas formed in either 1% LP (Lean_PD_) and 1% OP (Obese_PD_) and then re-dosed these particles, with their respective coronas, into cell cultures with either lean or obese serum (Supplementary Fig. 19). In agreement with our previous data, the obese plasma containing higher HDL levels clearly inhibited the uptake of LNPs with pre-formed HDL-enriched coronas. We also explored the effect of HDL supplementation on particles made with a variety of benchmark cationic lipids (CILs) and different lipid:mRNA (w:w) ratios (Fig. 6a). When human Huh7 hepatocytes were exposed to 1% lipoprotein deficient serum (LPDS) alone, LNP dosing resulted in much less eGFP expression at tested doses. A similar relationship between HDL and LNP particles was seen when HDL was added, with lower plateaus at lower mRNA doses and higher plateaus at higher mRNA doses. Again, further increasing the amount of HDL added inhibited cellular eGFP expression. In comparison, VLDL did not stimulate effective cellular eGFP expression using a similar range of particle numbers. The corona composition was determined for samples equivalent to the 100 ng/well dose (Fig. 6b). Remarkably, HDL and VLDL could both contribute ApoE to LNPcor, but otherwise the corona content was quite different. Apolipoprotein B (ApoB), another primary LDLr ligand in humans^40^, was highly enriched in VLDL coronas, and this increased when the number of VLDL particles were increased relative to the number of LNPs, indicating that while VLDL particles do interact with LNPs and can contribute to corona formation, this does not improve LNP function. In contrast, HDL particles containing ApoAII and ApoM had a potent effect on LNP function. HDL particles contributed-corona components at lower concentrations (2 × 10^7^ HDL/ well). This may be because the smaller HDL particles can interact more easily with LNP surfaces than the larger VLDL particles.

To gain further mechanistic insight into how corona composition affects nanoparticle uptake, LDLr and a widely recognized HDL receptor, Scavenger Receptor B1 (SRB1)^41^, were silenced with siRNA separately or simultaneously. The expression of LDLr and SRB1 by Huh7 hepatocytes was confirmed using Western Blot (Supplementary Fig. 20). Prior to LNP exposure, the cells were treated with the siRNA of interest for 48 h. LNPs were then dosed under the same conditions as described above. In the absence of HDL, inhibition of LDLr significantly reduced LNP uptake, while suppression of SRB1 did not affect uptake compared to control siRNA-treated cells. On the contrary, when LPDS was augmented with added HDL, silencing both receptors (alone or combined) decreased LNP uptake. This confirms that LDLr mediates general uptake of LNPs, while SRB1 was able to facilitate hepatocellular internalization of LNP with HDL-augmented corona specifically (Fig. 6c).

Finally, to validate our findings in a less artificial context, we investigated whether supplementing LNPs with HDL could improve their performance in vivo when there are physiological levels of plasma components in circulation, including HDL. Lean Zucker rats were injected with MC3 LNPs containing eGFP mRNA through the tail vein. The LNPs were either preincubated with additional HDL in 1% LPDS for 4 h prior to injection to form HDL-supplemented corona (HDL group) or, incubated with only 1% LPDS (Non-HDL group), as described in Supplementary Table 2. LNPs formulated with Poly Adenine (PolyA) mRNA were utilized as negative control (Neg ctrl group). 6 h after LNPs administration, the rats were sacrificed, and the expression of GFP in the liver, spleen and kidney was determined by ELISA, showing that LNPs with HDL-augmented coronas expressed more protein in the liver and less in other organs (Fig. 6d). Furthermore, supplementing LNPs with HDL caused a 4-fold increase in protein production in the liver compared to the Non-HDL group over the 6 h period. Without HDL preincubation, LNPs were also taken up more by other organs, presumably due to less complete liver uptake, suggesting that engineering particles to reduce HDL binding may be an effective strategy for promoting extrahepatic delivery.

These results show that HDL can improve the function of LNPs with different compositions and, that the improvements in productive delivery likely occur through mechanisms beyond the previously described ApoE-LDLr interactions, both in vitro and in vivo^42^.

## Discussion

Our results demonstrate that individual physiological states can affect LNP function through corona formation. The findings agree with previous literature that uses coronas formed around silica and graphene oxide nanoparticles as a sensitive tool for profiling disease-related biomarkers in human blood^43,44^. Overall, the heterogeneity amongst patients/diseases can hinder the success of clinical nanomedicines because most nanomedicine studies are carried out in unstratified patient populations^45^.

Our data also suggest that corona composition is more predictive for LNP efficacy than plasma biomarkers. For example, although animal 12 was classified as obese using relevant biomarkers, the plasma from this animal resulted in an LNP corona composition closer to lean plasma-derived corona and eGFP expression similar to the lean group. Our study is the first to show that important LNP engineering principles can be derived from stratified populations and, that unbiased corona analysis is essential for decoding the relationships between LNP components, corona composition, and LNP function.

Interestingly, we uncovered a relationship between how HDL is associated with the LNPcor, the amount of free HDL and, the cellular capacity for HDL-mediated uptake. At higher plasma concentrations, free HDL functioned antagonistically, whereas reduced plasma concentrations resulted in lower free HDL, greater particle uptake and, subsequently, improved mRNA expression. At the lowest lean plasma concentrations, there was not enough HDL to populate LNPcor, especially when using high doses of LNPs, degrading the LNP performance. The number of HDL particles is, however, elevated in obese rat plasmas, so the LNPs remained functional even at low obese plasma concentrations, and under these conditions, there was also less competition from unbound HDL. Augmenting LNP coronas with HDL tips the balance in favor of LNPs and allows them to compete with endogenous HDL particles for cellular receptors (such as SRB1) in-vivo. Taken together, these results are the first to indicate that LNP designs can be used to modulate LNP function and targeting by affecting how the particles interact with HDL in particular.

This study highlights the importance of HDL for LNP corona formation and LNP function both in vitro and in vivo. Most early studies accredited LNP delivery, especially hepatic delivery, to ApoE- mediated machinery. But for primarily analytical reasons, there was little understanding of the source of the ApoE. The lipid-bound state of ApoE^46^ and competition with non-corona ApoE^47^ from other sources may, however obscure the correlation with corona ApoE-induced uptake. We found that not only was corona ApoE content not the best predictor for LNP efficacy, but it was also negatively correlated with efficacy when the composition of the entire corona was considered. ApoE is important for the function of most LNPs^42^, but the context of the ApoE molecules is also important. The current study illustrates that among ApoE-containing lipoproteins, only HDL modulated LNP mRNA expression efficacy, even if other lipoprotein particles could contribute ApoE to LNPcor. It is not completely clear how exactly HDL particles enhance LNP function, but this is likely quite complicated and elucidating this will be the focus of future work.

The uniquely potentiating effect of HDL components in LNP coronas also means that in terms of corona composition, lipoproteins ApoM and/or ApoAII serve as better predictors of LNP efficacy. This also raises the question of how co-medication might affect the therapeutic efficacy of nanomedicines. While commonly used cholesterol-lowering drugs such as statins have a moderate stimulatory effect on HDL, Fibrate and prescription-strength Niacin (which can also be obtained in the everyday diet) can effectively increase HDL levels^48^ and this has implication for LNP dosing and performance.

In summary, we have created an efficient method for isolating LNPs, with intact coronas, from plasma, followed by an automated and unbiased mass spectrometric analysis of corona protein and lipid content. We show that the relationships identified can then be validated using in-vivo experiments rather than attempting to characterize in-situ corona composition definitively. This is advantageous as obese samples in particular, are very challenging to process analytically as they become more concentrated.

**Fig. 7:**
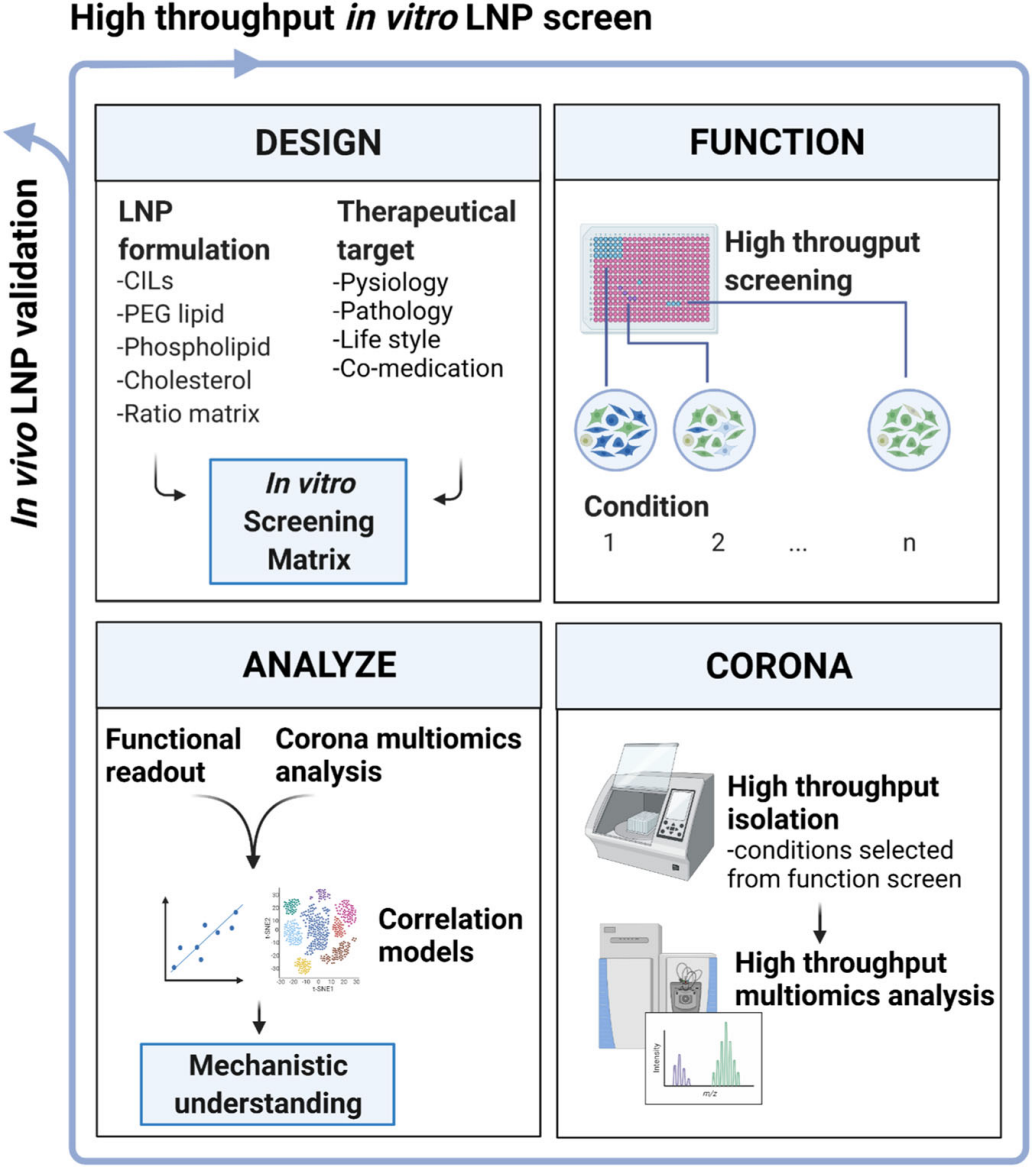
A scheme for combining high throughput LNP DMTA. Image-based screening and corona multiomics analysis reveal the relationships between efficacy and corona content. Functional changes following iterations in particle or cationic lipid (CIL) design can be identified and supplemented with corona multiomics data. This process can be repeated to obtain deeper mechanistic understanding, with in vivo validation of the resulting design concepts. Illustration is generated with BioRender.

Our study identified a lipoprotein fingerprint that promotes LNP function, leading us to examine the role of HDL-LNP interactions as a necessary factor for LNP efficacy. With these methods, it will now be possible to explore a greater variety of lipid nanoparticle formulations, biofluids, tissues, and physiological states, to explore the relationships between lipid nanoparticle corona content and delivery efficacy more comprehensively. While designing LNPs to promote particular corona compositions is a significant engineering challenge (workflow summarized in Fig. 7), the complexity of these interactions creates many opportunities for improving safety, reducing cost, targeting tissues and, tuning therapeutic particles for specific biological and pathobiological contexts.

## Methods

### Materials

Cholesterol (C8667), DSPC (LP-R4-076) was purchased from Merck. DOPE-Rhod (810150) was from Avanti Polar Lipids. DMPE-PEG2k (PM-020CN) was obtained from Nof America Corporation. Cationic ionizable lipids DLin-MC3-DMA, cKK-E12 and c12-200 were chemically synthesized in-house. Other chemicals, unless specified, were purchased from Merck and used as received. Additional materials are listed in context below.

### Formation of LNPs

Commonly used and clinically relevant reference LNPs containing DLin-MC3-DMA were formulated using a NanoAssemblr microfluidic mixer (Precision NanoSystems). As described in Supplementary Fig. 2, to achieve a 10:1 (w/w) lipid:mRNA ratio (N:P ratio = ~3:1), lipids were prepared in ethanol at a ratio of 50:38.5:9.9:1.5:0.1 (MC3:Cholesterol: DSPC:DMPE-PEG2000:DOPE-Rhod). Unlabeled and Cy5 labeled eGFP mRNA were prepared at a 4:1 ratio (TriLink:L7201/7701) in 50 mM citrate buffer (pH 3, TekNova: Q2445). Lipid and mRNA-containing solutions were mixed 1:3 (ethanol: citrate) at a constant flow rate of 12 ml/min to form LNPs. Formulated LNPs were dialyzed overnight in PBS (pH 7.4) at 4 °C. LNPs with other CILs were formulated in the same ratio of 50:38.5:9.9:1.5:0.1 (CILs:Cholesterol:DSPC:DMPE-PEG2000:DOPE-Rhod).

### Physical particle characterization

Particle size (Z-average diameter) and polydispersity index (PDI) were determined by dynamic light scattering utilizing a Malvern Zetasizer ZS for newly formulated LNPs by NanoAssemblr or a Malvern Zetasizer APS for LNPs and harvested LNPcor. Following formulation, the LNP surface zeta-potential was measured by a Malvern Zetasizer ZS. In both cases, viscosity and refractive index values, 0.8872 mPa and 1.33 respectively, were used for data analysis (Zetasizer software, v7.12).

The mRNA concentration and encapsulation of LNPs were evaluated by Ribogreen dye (Thermo Fisher Scientific) according to the manufacturer’s guidelines. ±1% Triton was used to ascertain the fraction of encapsulated mRNA by comparison to a relevant free mRNA standard curve.

Particle number and size distribution of LNPs, coronated LNPs and, endogenous particles in blood plasmas were determined by nanoparticle tracking analysis (NTA). During each NTA measurement, a movie of particles in Brownian motion was recorded (Supplementary movie 1–6). The analysis was performed at RT using a Nanosight LM14 model (Nanosight, UK) equipped with a blue laser (405 nm, 70 mW) and SCMOS camera. The samples were prepared by diluting stocks from 1:2000 to 1:10,000 in PBS before analysis to obtain an appropriate concentration for the NTA measurements. Three 90-s videos were recorded. The data analysis was performed using the NTA software v3.0.

### Cell culture

H4-II-E-C3 (CRL-1600), McA-RH-7777 (CRL-1601), and NRK-49F (CRL-1570) cells were purchased from ATCC. Huh7 (Riken - RCB1366) cells were a kind gift from Samir El-Andaloussi (KI, Stockholm). All cell lines were authenticated by STR profiling and tested negative for mycoplasma contamination prior to cryopreservation and local cell banking. Cells were maintained in an incubator at 37 °C with humidity in the complete media (DMEM + Glutamax supplemented with 10% fetal bovine serum).

### Animals

Ten-week-old male lean and obese Zucker rats (Crl:ZUC-Lepr^fa^) were purchased from Charles River Laboratories (Maryland, USA) and group-housed (*n* = 4/cage) in an Association for Assessment and Accreditation of Laboratory Animal Care (AAALAC) accredited facility at 20–22 °C and relative humidity of 40–60% with a 12-h day/night cycle. The rats had free access to water and a standard rodent chow diet (R70, Lactamin AB). At 20 weeks of age, the rats fasted for 4 h, and a tail vein blood sample was obtained for glucose (Accu-Chek® Mobile, Roche Diagnostics) and glycosylated hemoglobin (HbA1c, PTS Diagnostics) analyses. Thereafter, the rats were euthanized using isoflurane anesthesia (Forene®, Abbott) and blood was collected from the heart and EDTA plasma was isolated and stored at −20 °C. The experimental procedures were approved by the local Ethics Committee for Animal Experimentation (Gothenburg region, Sweden).

### Blood plasma analysis

Plasma insulin levels were measured using a mouse/rat insulin kit (#K152BZC-1, Meso Scale Discovery). Plasma triglyceride (#11877771, Roche Diagnostics), cholesterol (#A11A01634, Horiba Medical) and alanine aminotransferase (ALT, #A11A01627, Horiba Medical) levels were analyzed using an ABX Pentra 400 instrument (Horiba Medical).

### Imaging experiments and quantification

Cells were seeded at appropriate densities into CellCarrier-384 Ultra plates (#6007558, PerkinElmer) in complete media a minimum of 16 h prior to treatment. At the experimental start, the media on cells was removed and replaced with media containing the experimental treatment as denoted in the relevant figures. The lean and obese rat plasma used were withdrawn as described above and characterized as shown in Supplementary Fig. 2 and Data 1. The human lipoprotein deficient serum (#LP4, Merck), HDL (#LP3, Merck), VLDL (#LP1, Merck), and CM (#SPR6304, Merck) were commercially available.

Following a 1 h incubation at 37 °C with 100% humidity, the cell plate was then imaged. Live-cell imaging was carried out with a CV7000 (Yokogawa) spinning disk confocal microscope utilizing a 20× objective (NA 0.75) in a humidified chamber maintained at 5% CO_2_. Images were obtained using a bright-field lamp (for digital phase contrast, DPC) and the following fluorescence excitation (emission) wavelengths: 488 nm laser (BP522/35), 561 nm laser (BP600/37) and 640 nm laser (BP676/29). For time-resolved measurements, the same fields of view were imaged over time with three optical planes (slicing interval: 3 µm). Final fluorescent image stacks were constructed using maximum intensity projections. Images were processed and analyzed, for relevant features and parameters indicated in figures, utilizing Columbus image-analysis software (Perkin Elmer, v2.9.0). Briefly, cells were identified using digital phase contrast and the ‘find cells’ building block (Method M) within Columbus software that identifies individual cell boundaries. Within individual cell regions of interest, fluorescent intensities were quantified for each relevant fluorophore. Data were exported and plotted with JMP (v15.0.0) and Graphpad Prism (v9.0.0) with appropriate statistical analysis. The total amount of eGFP produced over the 10 h time course was summarized as a new parameter termed 10 h area under the curve (AUC_10h_) for OPLS correlation analysis.

### LNPs stability in culture medium

0.8 µg mRNA containing LNPs were resuspended in a final volume of 200 µL using different rat plasmas or PBS. 50 µL of these suspensions were pipetted in a 384 well plate (Greiner, #781209) in triplicates, and the plate was incubated for 0 h, 4 h and 8 h at 37 °C. At these timepoints, the emission spectra were obtained using a Safire II plate reader (Tecan) and 560 nm excitation with emission scanning from 570 to 750 nm. FRET efficiency is calculated as the ratio between acceptor molecule (Cy5) emission peak (FA) and the ratio of Rhod (FD) and Cy5 emission in total (Eq. 1).

1 $${{{{{\rm{FRET}}}}}}=\frac{{{{{{\rm{FA}}}}}}*}{{{{{{\rm{FD}}}}}}*\ast+{{{{{\rm{FA}}}}}}}$$

*The emission peak for Cy5 (FA) was the maximum RFU measured for 668–678 nm.

**The emission peak for Rhodamine (FD) was the maximum RFU measured for 586–594 nm.

### Asymmetric flow field-flow fractionation (AF4) separation

AF4 separation was performed using an Agilent 1260 Infinity HPLC (Agilent) connected to an Eclipse AF4 separation system (Wyatt Technology) followed by a Dawn Heleos-II 18-angle MALS detector (Wyatt Technology). Separations were performed using a 10-kDa molecular-mass cutoff polyether sulfone membrane with an S-350 μm spacer in a 153 mm separation channel. Samples were introduced to the channel at an inlet flow of 0.2 mL/min and subsequently focused at the head of the channel at a focus flow rate of 1.5 mL/min. Samples were eluted over 25 min with a channel flow rate of 1 mL/min and a cross-flow gradient of 3.0–0 mL/min.

### LNP corona isolation

LNPs were incubated in cell culture media supplemented with 1% individual plasmas at 200 ng mRNA LNPs dose (4 µg/mL of mRNA). M-270 Epoxy Dynabeads (#14321D, Thermo Fisher Scientific) were cross-linked with monoclonal anti-PEG [PEG-2-128] (Abcam) according to the manufacturer’s instructions. A KingFisher Flex magnetic purifier (Thermo Fisher Scientific) with 96 magnetic rod heads was used to separate the free-proteins, endogenous nanosized particles from the LNPcor. Briefly, the incubation, wash, and elution procedures were performed using the optimized conditions indicated in Supplementary Fig. 10. The antibody-conjugated Dynabeads were incubated with LNPs within media for 20 min at an antibody: mRNA (wt:wt) ratio of 1 with gentle mixing. At the end of incubation, the Dynabeads were extracted using magnet rods and washed four times with PBS. A basic pH elution buffer containing 0.5 M NH_4_OH and 0.5 mM EDTA was utilized to release LNPcor from Dynabeads. LNP pull-down quantification was performed by Cy5 fluorescence readout.

### Cryo-electron microscopy

For cryo-electron microscopy experiments, lean and obese LNP samples at a concentration of ~10^13^ particles/ml were incubated with glow discharged carbon-coated copper grids (SPI supplies), following vitrification at 10 °C and 99% humidity by using a Leica EM GP automatic plunge freezer (Leica Microsystems Company). The excess sample was removed by blotting dry the grid for 2.5 s with filter paper and plunging it into liquid ethane at −180 °C. Following vitrification, grids were stored immersed in liquid nitrogen until use. Before imaging, the grids were mounted in a Gatan 626 cryo-holder (Gatan Company) and analyzed using an FEI Tecnai G2 Spirit BioTwin transmission electron microscope (Thermo Fisher Scientific). Pictures were taken with a Morada digital camera (Olympus Soft Image Solutions) and iTEM TIA image capture software (v4.7, Olympus).

### Proteomics analysis

Corona protein digestion was performed on recovered LNPs from individual plasmas containing an equal amount of mRNA. Briefly, sample denaturation and reduction were performed using a 30 min one-step 8 M urea (#U1250, Merck) and TCEP bond-breaker solution (#77720, Thermo Fisher Scientific), followed by a 30 min alkylation step using a 2-chloroacetamide reagent (#22790, Merck). Protein digestion was done overnight in trypsin (#EMS0004, Merck) and ceased by the addition of formic acid. Digestions were measured using a Q-Exactive HF mass spectrometer (Thermo Fisher Scientific) coupled with an Evosep One (Evosep) automatic sample loader equipped with Evotip disposable C18 trap columns (Evosep) for in-line peptide desalting and purification immediately prior to analytical column separation with a preset, 30 samples per day (30-SPD), loading sequence. Briefly, purified peptides were separated on an 8 cm analytical reverse-phase column (Evosep) with gradient off-set focusing to achieve a 3–40% acetonitrile within a 44 min loop at a 0.5 µL/min flow rate.

MS raw files were analyzed by MaxQuant software (v1.6.6.0). Proteins were identified using the Uniprot FASTA database (Rattus norvegicus UP000002494, Homo sapiens UP000005640, where applicable) with N-terminal acetylation and methionine oxidations as variable modifications, and cysteine carbamidomethylation as a fixed modification. The false discovery rate (FDR) was set to 1% by reverse database search for both proteins and peptides with a minimum length of seven amino acids. Enzyme specificity was set as trypsin (cleavage at C-terminal to arginine and lysine). A maximum of two missed cleavages was allowed in the database search. Peptide identification was performed with an initial precursor mass deviation tolerance up to 6 ppm and a main mass deviation tolerance to 20 ppm. Matching between runs was performed among samples. Proteins matching to the reversed database were filtered out. For protein quantification, MaxQuant computes raw protein intensities as the sum of all identified peptide intensities. Label-free quantification (LFQ) and intensity-based absolute quantification (iBAQ) were calibrated from raw protein intensities with a minimum peptide ratio count of 1.

To compute protein relative abundance (PRA), intensity-based absolute quantification (iBAQ) obtained from raw protein intensities were divided by the number of theoretically observable peptides calculated using in silico protein digestion. Then, iBAQ intensities of identified protein were used to calculate PRA.

To compare each identified protein between samples, statistical analyses were first performed with the Perseus software (v1.6.2.3) using LFQ intensities. A valid value threshold was first applied to identify significantly altered corona proteins among all identified proteins. Only proteins identified within all conditions and replicates, followed by a multi-sample ANOVA test, are considered valid hits (significance cutoff: permutation-based FDR < 5%). Further hierarchical clustering analysis was performed with JMP (v15.0.0). OPLS analysis was performed with SIMCA (v16.0.1), using z-score normalized LFQ values. The Pearson correlation between single corona component and cellular eGFP was carried out with Prism GraphPad (v9.0.0). The correlation coefficient r was controlled by nonparametric (Spearman) correlation *P* value.

### Targeted lipidomics analysis

The molecular species of corona phospholipids was extracted with the 2-phase BuMe system method^33^. Briefly, the samples were combined with a BuMe mixture (butanol: methanol ratio=3:1). SPLASH I S(#330707, Avanti polar lipids) was included in the BuMe as an internal standard (added prior extraction). pH adjustment (when applicable) was carried out with HAc. Following a series of centrifugations, the extracted lipids were then evaporated and redissolved in MeOH and analyzed by HILIC-UPLC-ESI-MS/MS. The UPLC-MS/MS system was an Acquity I class coupled to a Waters Xevo TQ-S triple quadrupole mass spectrometer with a BEH amide analytical column (#186004801, Waters) with Waters Masslynx 4.1 Software. The analytical column was a BEH amide, 100 × 2.1 mm, 1.7 µm particle size (#186004801, Waters)

The mobile phase A was composed with 95% Acetonitrile and 5 mM Ammonium Formate. The mobile phase B was 10 mM Ammonium Formate (#17843, Honeywell). The gradient was from 1% B in A, held for 1 min, and then to 30% B in A in 6 min, back to 1% B in 0.1 min and allowed to reequilibrate for 3 min. The flow was 0.4 ml/min and injection volume was 2 µl.

The mass spectrometer was operated in positive ESI mode with a source temp of 150 °C, a desolvation temp of 600 °C, a cone gas flow of 150 L/h, a Desolvation gas flow of 1200 L/h and a collision gas flow of 0.15 mL/m. MS2 (QQQ, low resolution) was used for identification with 1 Da total isolation window for precursor ion isolation. 29 transitions were monitored for PE and 11 for LPE, all transitions having a neutral loss of 141 Da. Twenty-nine transitions were monitored for PC, 13 for SM, and 20 for LPC all transitions having a common 184 Da product ion. The Cone voltage was kept at 30 V for all transitions and the collision energy was kept at 25 V for all transitions. Injection blanks were employed between samples. Lipid quantification and identification data was reported as the raw output (included in the supplementary).

### Dot blotting

2 µL solution containing LNPcor or corresponding control samples was spotted to Nitrocellulose membranes (#LC2001, Thermo Fisher Scientific). Membranes were blocked with Intercept (TBS) blocking buffer (LI-COR) for 30 min at RT and incubated with primary antibodies Cy5 (#ab52061, Abcam), PEG (#ab51257, Abcam), Apo AII (#ab92478, Abcam), Apo CII (#ab230447, Abcam), Apo CIII (#ab76305, Abcam) and ApoE (#ab183597, Abcam) where appropriate, diluted 1:1000 in blocking buffer at for 30 min at RT. Membranes were washed three times with 0.1% TBS-Tween and incubated for 30 min at RT with IRDye 680RD Goat anti-Mouse IgG (#926-68070, LI-COR Biotechnology) and 800CW goat anti-rabbit IgG (#926-32211, LI-COR Biotechnology) diluted 1:2000 in 0.1% TBS-Tween. Following three washes, membranes were visualized with the Odyssey CLx imaging system and processed in Image Studio (v4.0, LI-COR).

### Receptor silencing

The silencing of Huh7 cell surface receptors was conducted, according to the manufacturer’s instructions, using siRNA for LDLr (#s224007, Thermo Fisher Scientific), SRB1 (#s2648, Thermo Fisher Scientific), and a scrambled sequence as negative control (#4390843, Thermo Fisher Scientific) with the reverse transfection method. In general, the siRNA of interest, as described in Fig. 6c and Supplementary Fig. 20, was mixed with Lipofectamine™ RNAiMAX (#13778100, Thermo Fisher Scientific) in OptiMEM (#11058021, Thermo Fisher Scientific) and incubated at RT according to the manufacturer’s instructions with a final concentration of 1 pmol/well. Huh7 hepatocytes were then seeded the siRNA/Lipofectamine complexes. For imaging evaluation of LNP cellular uptake and receptor expression evaluation, cells were prepared in 384 well and 24 well formats, respectively.

### Western blotting

48 h after dosing with the siRNA against the receptors of interest, the cells were lysed with RIPA buffer (#89900, Thermo Fisher Scientific) supplemented with proteinase inhibitor (#11836153001, Merck) and incubated for an additional 30 min at 4 ^o^C. The cellular proteins were then analyzed using NuPAGE™ 4–12% Bis-Tris gel (NP0323, Thermo Fisher Scientific) and transferred using PVDF membranes (#1704156, Bio-Rad). Membranes were blocked with Intercept (TBS) blocking buffer (LI-COR) for 60 min at RT and incubated with primary antibodies against LDLr (#ab30532, Abcam), SRB1 (#ab217318, Abcam), and/or Histone H2A (#ab18255, Abcam), where appropriate, all diluted 1:2000 in blocking buffer at for overnight at 4 ^o^C. Membranes were washed three times with 0.1% TBS-Tween and incubated for 60 min at 800CW goat anti-rabbit IgG (#926-32211, LI-COR) diluted 1:5000 in 0.1% TBS-Tween. Following three washes, membranes were imaged using the Odyssey CLx imaging system and followed by processing using Image Studio (v4.0, LI-COR).

### eGFP ELISA

6 h after the administration of different treatments as indicated in Supplementary Table 2, eGFP levels were measured with a GFP ELISA Kit (#ab171581, Abcam) according to the manufacturer’s instruction. Briefly, the liver, spleen and kidney tissues were homogenized using a Precellys tissue homogenizer (Bertin Corp.) in the extraction buffer offered by the kit. An appropriate amount of tissue lysate was transferred into ELISA detect plate, and the OD was measured with a Pherastar plate reader (BMG Labtech) at 450 nm. The level of eGFP in the negative control sample was below the limit of detection.

### Statistics and reproducibility

Statistical testing was carried out with relevant multiple comparisons and post-testing where appropriate as indicated in the respective methods. For all experiments, no data were excluded from the analysis and all reported results were replicable. For imaging experiments, statistical testing was carried out upon three independent experimental replicates. For omics experiments, a minimum number of two independent experimental replicates were used for data statistics. See figure legends for full details of replicates, statistical testing, and significance. For further multivariate analysis information, please see the proteomics analysis section and the main text.

### Reporting summary

Further information on research design is available in the Nature Portfolio Reporting Summary linked to this article.

### Supplementary information

Supplementary Information

Description of Additional Supplementary Files Document

Supplementary_Data_1

Supplementary_Data_2

Supplementary_Data_3

Supplementary Movie 1

Supplementary Movie 2

Supplementary Movie 3

Supplementary Movie 4

Supplementary Movie 5

Supplementary Movie 6

Reporting Summary

### Source data

Source Data

## Data Availability

The datasets generated during and/or analyzed during the current study are provided in the Source Data file and Supplementary Information. The proteomics raw files were searched against UniProt FASTA database (Rattus norvegicus UP000002494, www.uniprot.org/taxonomy/10116; Homo sapiens UP000005640, www.uniprot.org/taxonomy/9606, where applicable). Data generated in this study have been deposited in the PRIDE Archive under accession code PXD041925, PXD041938, and PXD041944. Source data are provided with this paper.
