# Supplementary material for: Multiomics analysis of naturally efficacious lipid nanoparticle coronas reveals high-density lipoprotein is necessary for their function
Source: Nat Commun. 2023 Jul 6;14:4007. doi: 10.1038/s41467-023-39768-9 (PMC10325984; doi:10.1038/s41467-023-39768-9)
Supplement: Supplementary file 1 — Supplementary Information [file 41467_2023_39768_MOESM1_ESM.pdf]

Supplementary Information

Multiomics analysis of naturally efficacious lipid nanoparticle coronas reveals high-density lipoprotein is necessary for their function.

Liu, K et al.

- Supplementary Figures 1-20.
- Supplementary Tables 1-2.

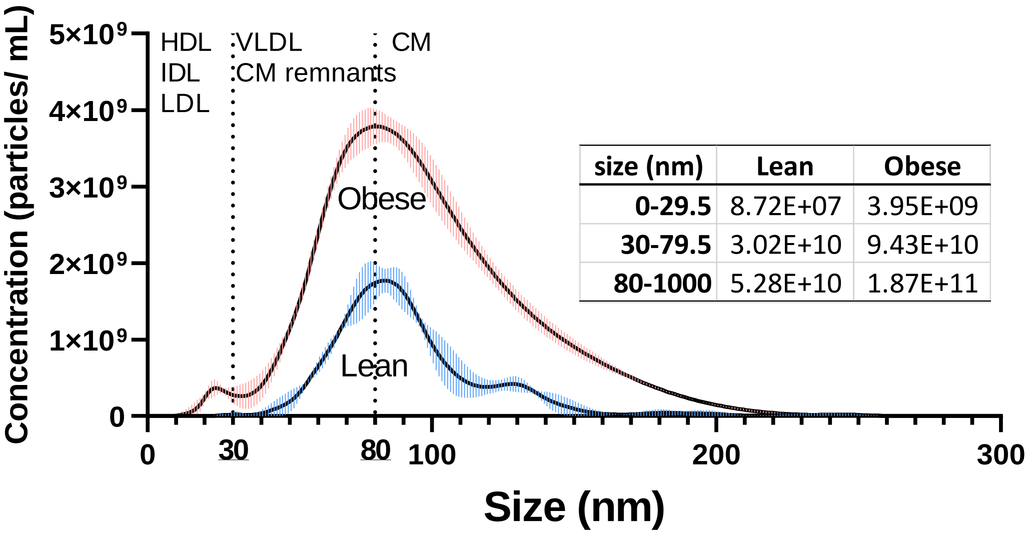

size (nm)	Lean	Obese
**0-29.5**	8.72E+07	3.95E+09
**30-79.5**	3.02E+10	9.43E+10
**80-1000**	5.28E+10	1.87E+11

Supplementary Fig. 1. The size profile of endogenous particles in plasma are largely overlapped with LNP (~80 nm). The size and distribution of particle like events were characterized by nanoparticle tracking analysis (NTA) in LP and OP plasmas. The generally accepted size range cut-off of lipoprotein species, indicated by dotted lines, is shown at the top of the figure. Black solid lines: mean particle count. Blue and red coloured bands: standard deviation (n=3 experimental replicates). The table shows the particular events number counting within each size range. HDL: high-density lipoprotein; IDL: intermediate-density lipoprotein; LDL: low-density lipoprotein; VLDL: very low-density lipoprotein; CM: chylomicron. Source data are provided as a Source Data file.

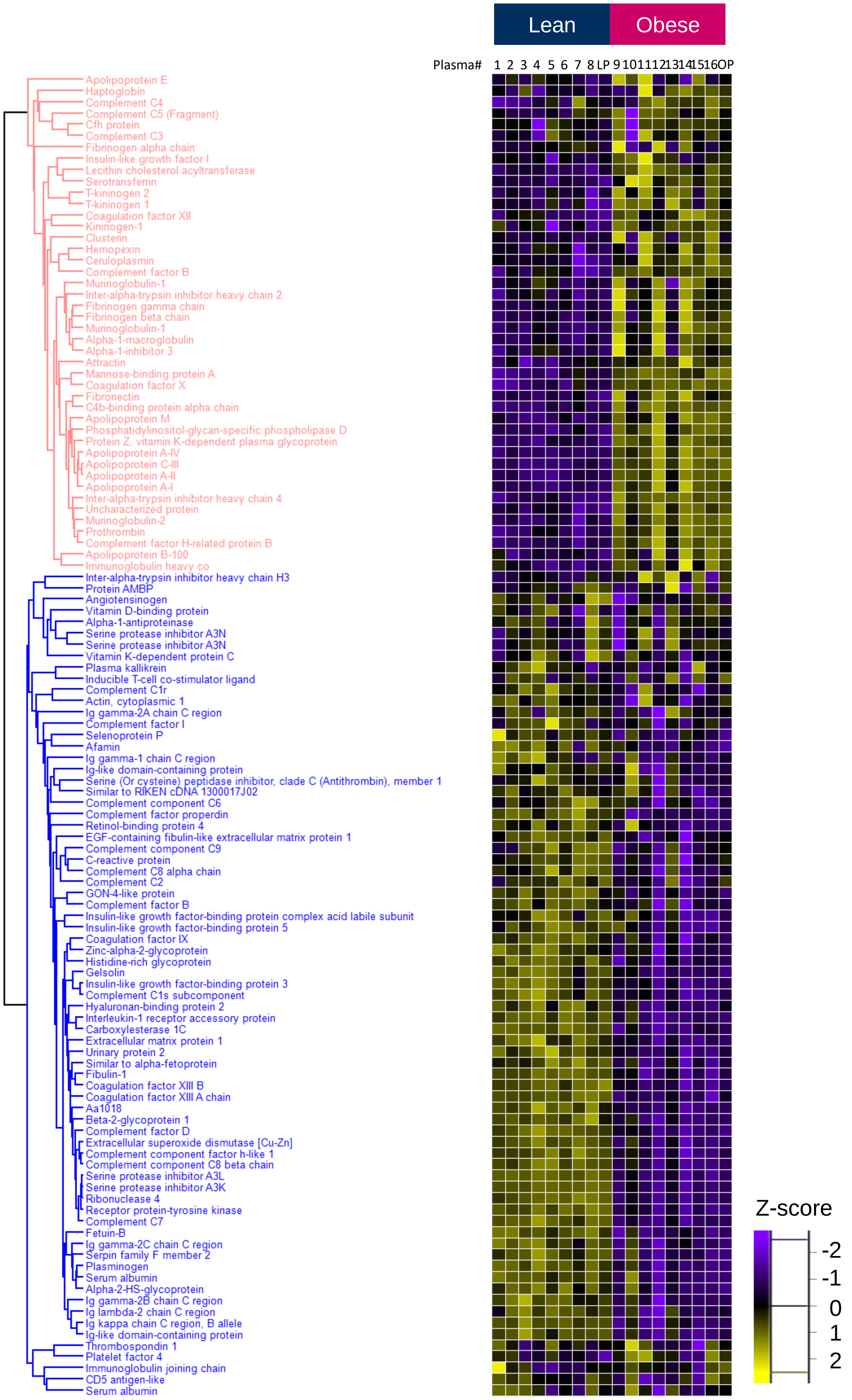

Supplementary Fig. 2. The proteomics profile of individual plasmas. The plasma protein expression was normalized with Z-scoring (n=2). Differentially expressed proteins were identified by ANOVA (FDR<5%). The red cluster represents proteins upregulated in obese plasmas. The blue cluster represents proteins upregulated in lean plasmas.

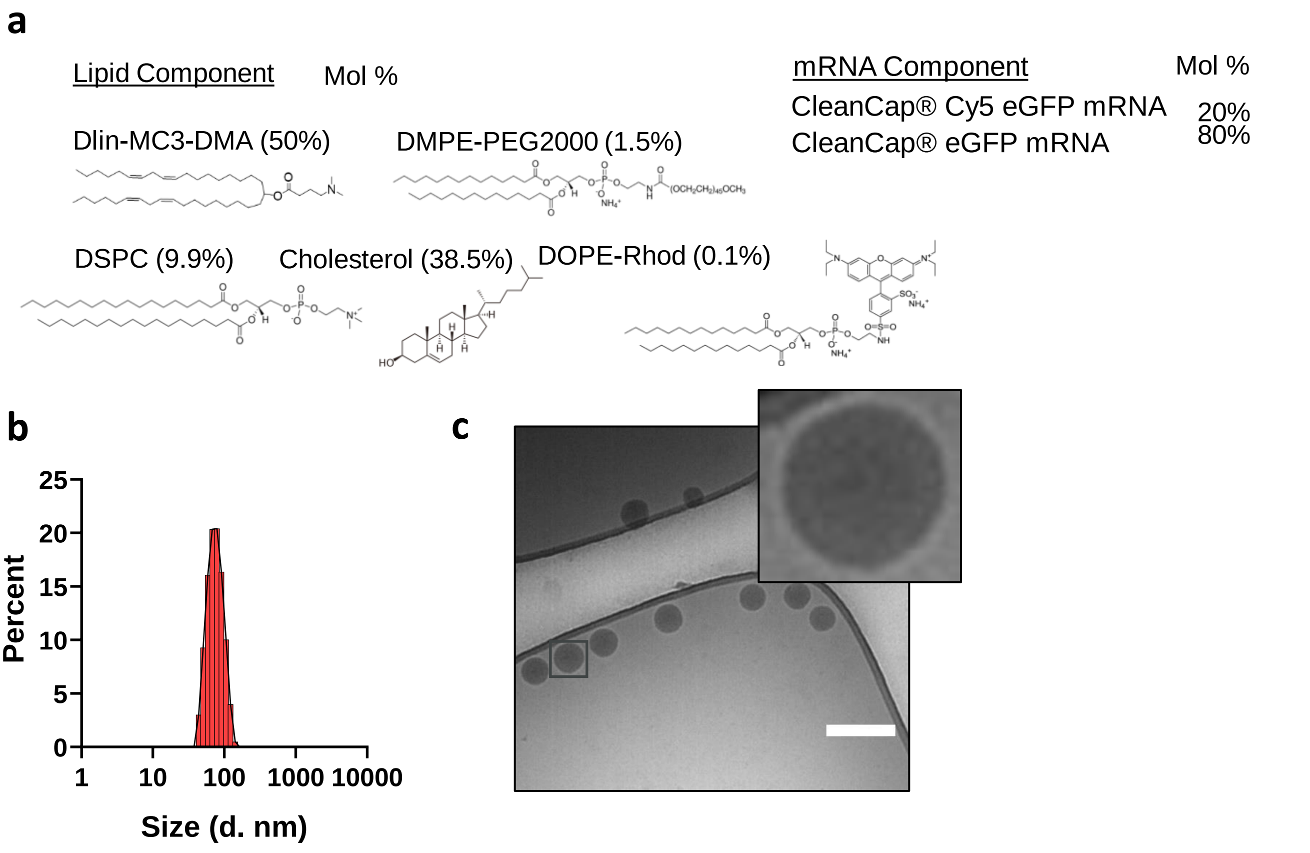

Supplementary Fig. 3. The LNPs formulation and characterization.
a, The composition of LNPs used in this study. Dlin-MC3-DMA: dilinoleylmethyl-4-dimethylaminobutyrate, DSPC: distearoylphosphatidylcholine , DMPE: dimethylphosphatidylethanolamine, DOPE: dioleoylphosphatidylethanolamine, PEG: polyethylene glycol.
b, A representative size distribution of LNPs measured by dynamic light scattering (DLS) from a single LNP batch. The size of each LNP batch used in this study were tested.
c, Representative LNP Cryo-EM morphology (scale bar=200 nm) selected from 20 images taken from one LNP batch

Supplementary Table 1. The characterization of LNPs batches used in this study.

Batch	Size (d.nm)	PDI	Z-potential (mV)	Encapsulation (%)
1	80.4	0.032	-14.0	98.3
2	77.5	0.140	-13.7	98.3
3	81.4	0.043	-14.5	98.0

*PDI: polydispersity index

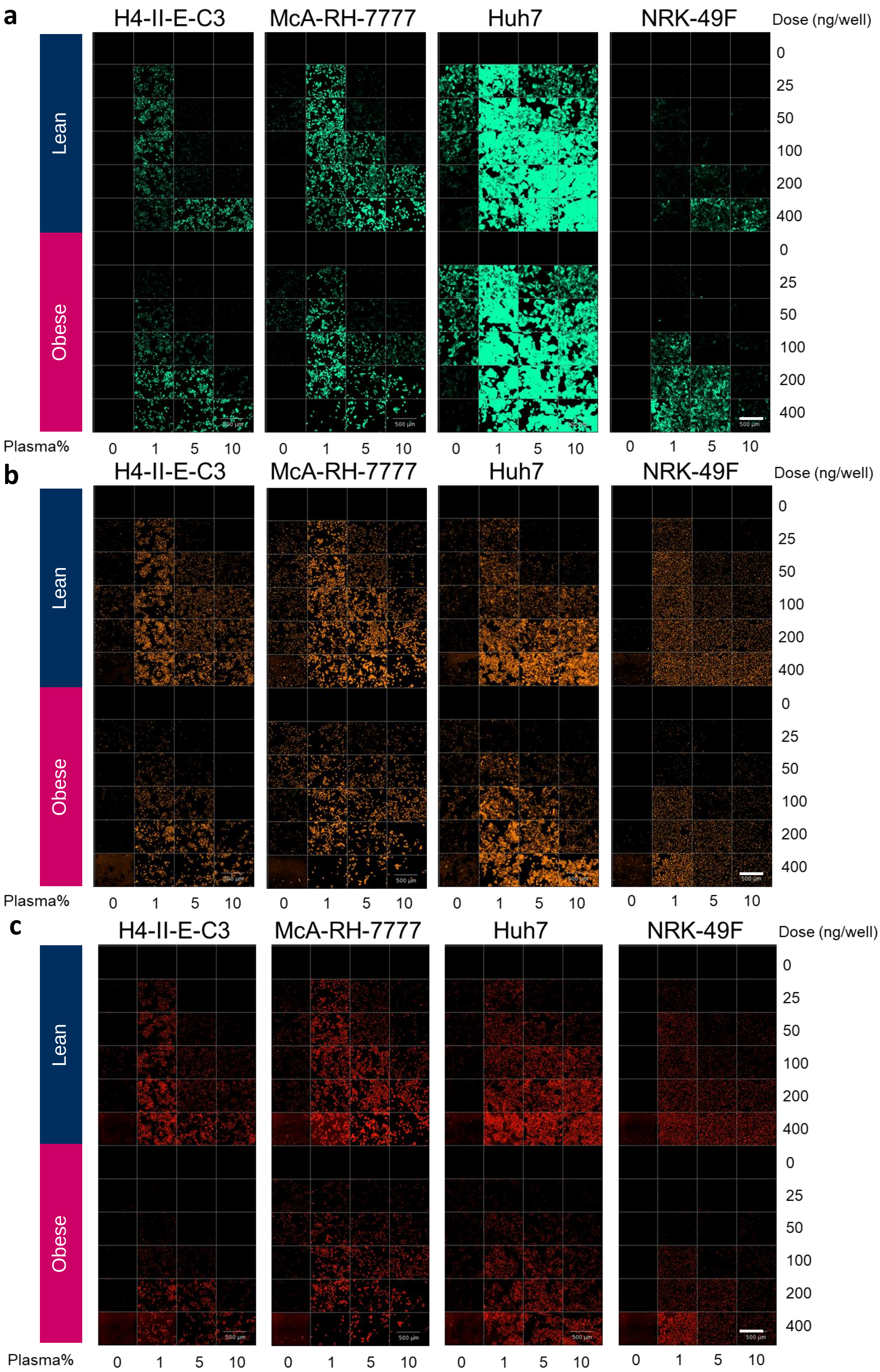
a
H4-II-E-C3
McA-RH-7777
Huh7
NRK-49F
Dose (ng/well)
Lean
Obese
0
25
50
100
200
400
Plasma%
0
1
5
10
b
c
500 μm

Supplementary Fig. 4. Panel of representative high throughput confocal images at 10h timepoint.
a, The eGFP mRNA expression
b, the uptake of Rhodamine labelled LNP lipids
c, the uptake of LNP-encapsulated cyanine-5-labelled mRNA
Representative images from three experiment replicates. Quantitative well-level data was calculated by averaging measurements of individual cells and this is shown in figure 1. Scale bars=500 µm. Note that the dynamic range of the data is not possible to accurately convey here, so these figures are provided as qualitative data. Please refer to Fig. 1 and supplementary Fig. 5 for more quantitative data.

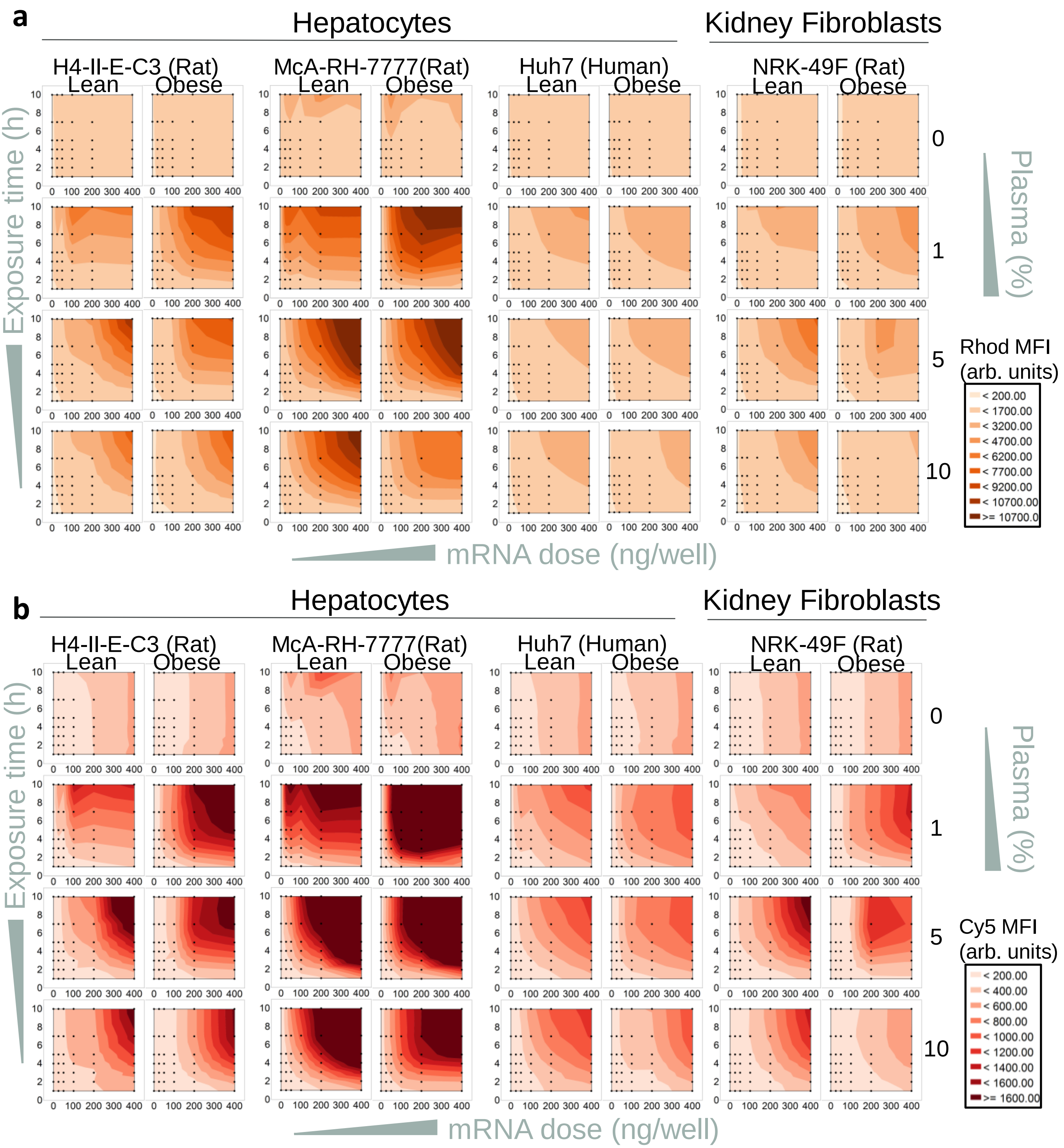

Supplementary Fig. 5. The cellular LNP uptake. A series of mRNA doses (25-400 ng/well; 0.5-8 µg/ml)) were tested in the presence of LP or OP plasma (0, 1, 5 and 10%) with H4-II-E-C3, McA-RH-7777, Huh7 and NRK-49F cell lines over 10h. The cellular mean fluorescent intensity (MFI) was extracted from raw microscopic images and summarized in the contour map (black dot, n=3) for Rhod labelled lipid (**a**) and Cy5 labelled mRNA (**b**). The corresponding cellular eGFP expression is demonstrated in Fig. 1b. Source data are provided as a Source Data file.

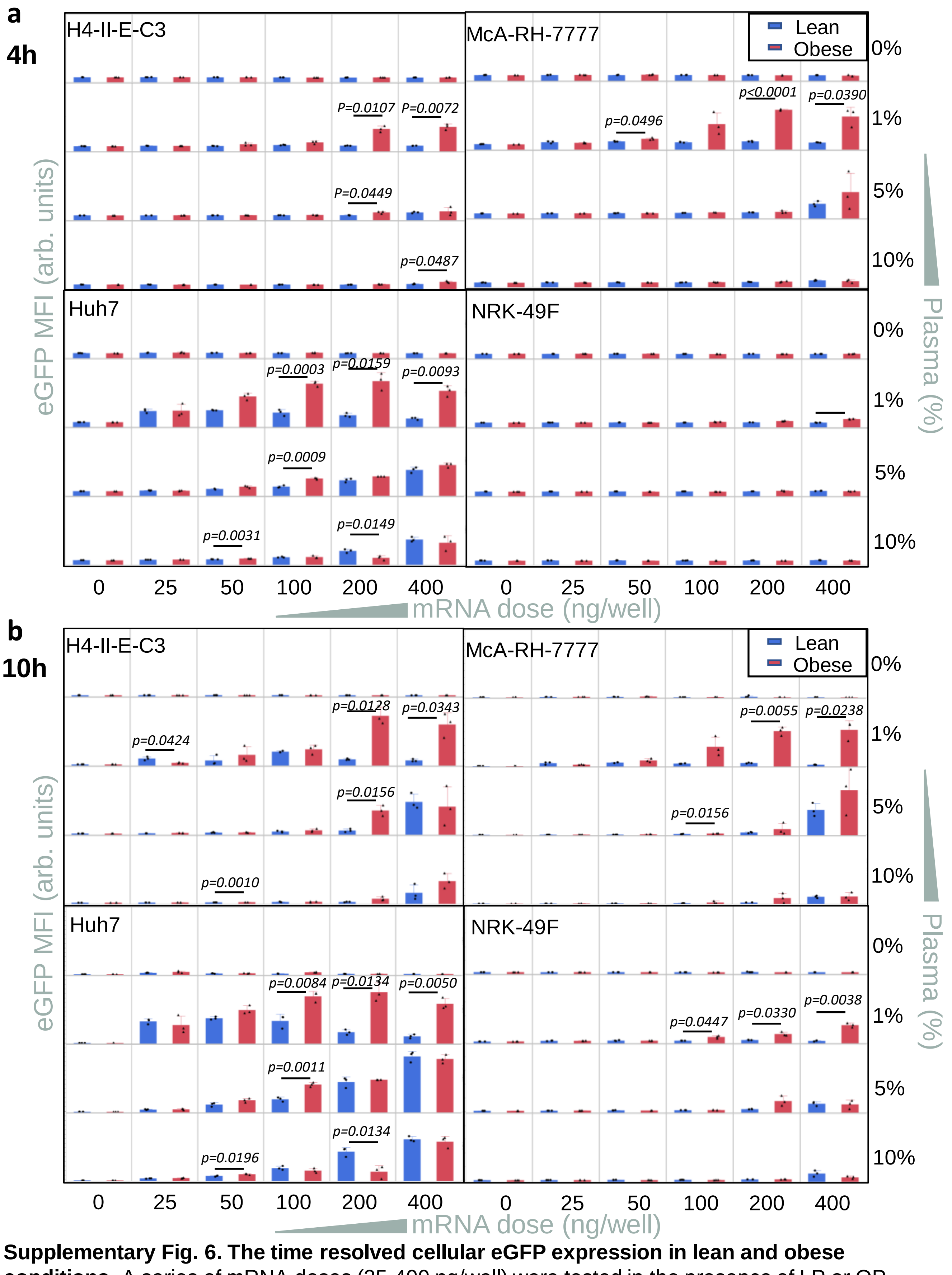

Supplementary Fig. 6. The time resolved cellular eGFP expression in lean and obese conditions. A series of mRNA doses (25-400 ng/well) were tested in the presence of LP or OP plasma (0, 1, 5 and 10%) *in vitro.* The cellular eGFP fluorescent intensity (MFI) was quantified. **a**, The difference between LP and OP conditions started at 4h. **b**, The difference persisted until 10h time point. The error bars represent standard deviation of the mean values derived from raw images (n=3 experimental replicates). Significance P values are determined by unpaired two-tailed t-test. Source data are provided as a Source Data file.

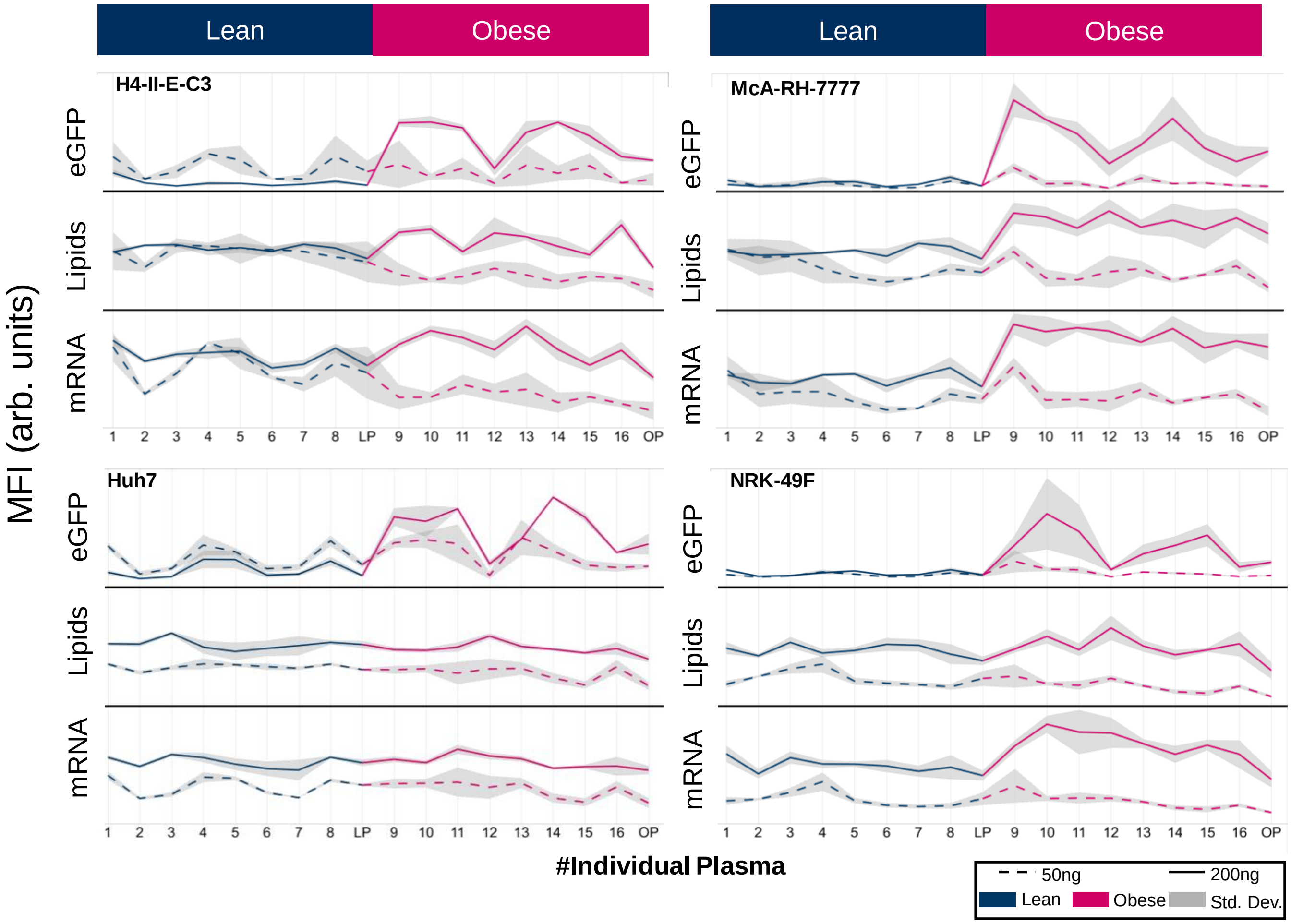

Supplementary Fig. 7. The individual variation of LNPs mRNA delivery efficacy. The LNP cellular uptake and cargo mRNA delivery were measured using high throughput confocal imaging with each individual rat plasma. Two mRNA doses (50 and 200 ng/well, dashed and solid lines respectively) were tested together with 1% plasma supplementation. The cellular mean fluorescent intensity (MFI) was quantified using image analysis and summarized for cellular eGFP, Rhod and Cy5 intensity. The mean is indicated by the central line for lean individuals (navy) and obese individuals (ruby). The standard deviation was indicated by the grey area. The error band represent standard deviation of the mean values derived from raw images (n=3 experimental replicates). Source data are provided as a Source Data file.

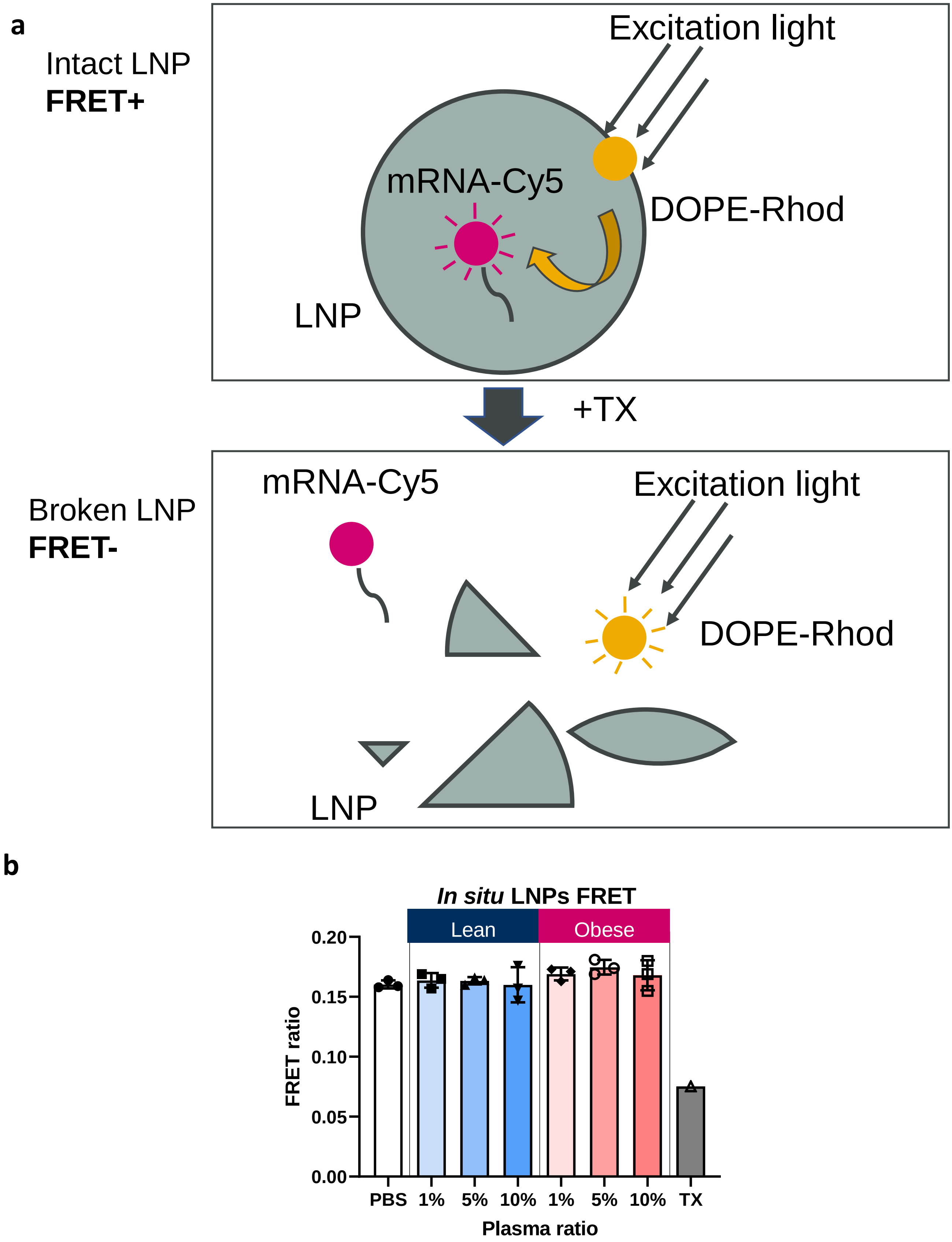

Supplementary Fig. 8. The FRET profile of LNPs.

a, A schematic diagram illustrate the mechanism of LNP integrity FRET measurement. The DOPE-Rhod and mRNA-Cy5 stay in close proximity to have FRET effect. When LNP integrity is compromised, the extended distance between mRNA and DOPE disables FRET effect.

b, The FRET profiles were measured post 4h incubation in 1-10% of lean pool and obese pool plasma. TX: 1% Triton X-100. The error bars represent standard deviation of the mean values derived from experimental replicates (n=3). Source data are provided as a Source Data file.

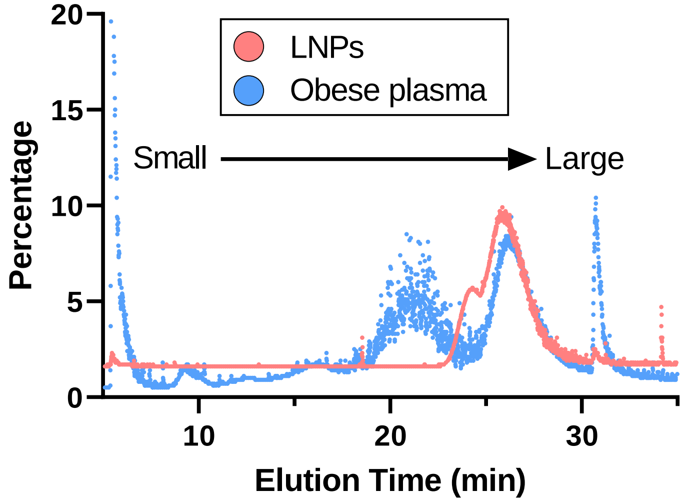

Supplementary Fig. 9. The elution profile of LNPs and endogenous plasma particles by asymmetric flow field-flow fractionation (AF4). The time-resolved elution profile was normalized to the total signal intensity of the overall time course. The size and density of LNPs used in this study overlap with endogenous lipoprotein particles, especially very-low-density lipoproteins (VLDLs), leading to contamination of the LNP-corona populations with endogenous lipoprotein particles and potentially extracellular vesicles as well. Source data are provided as a Source Data file.

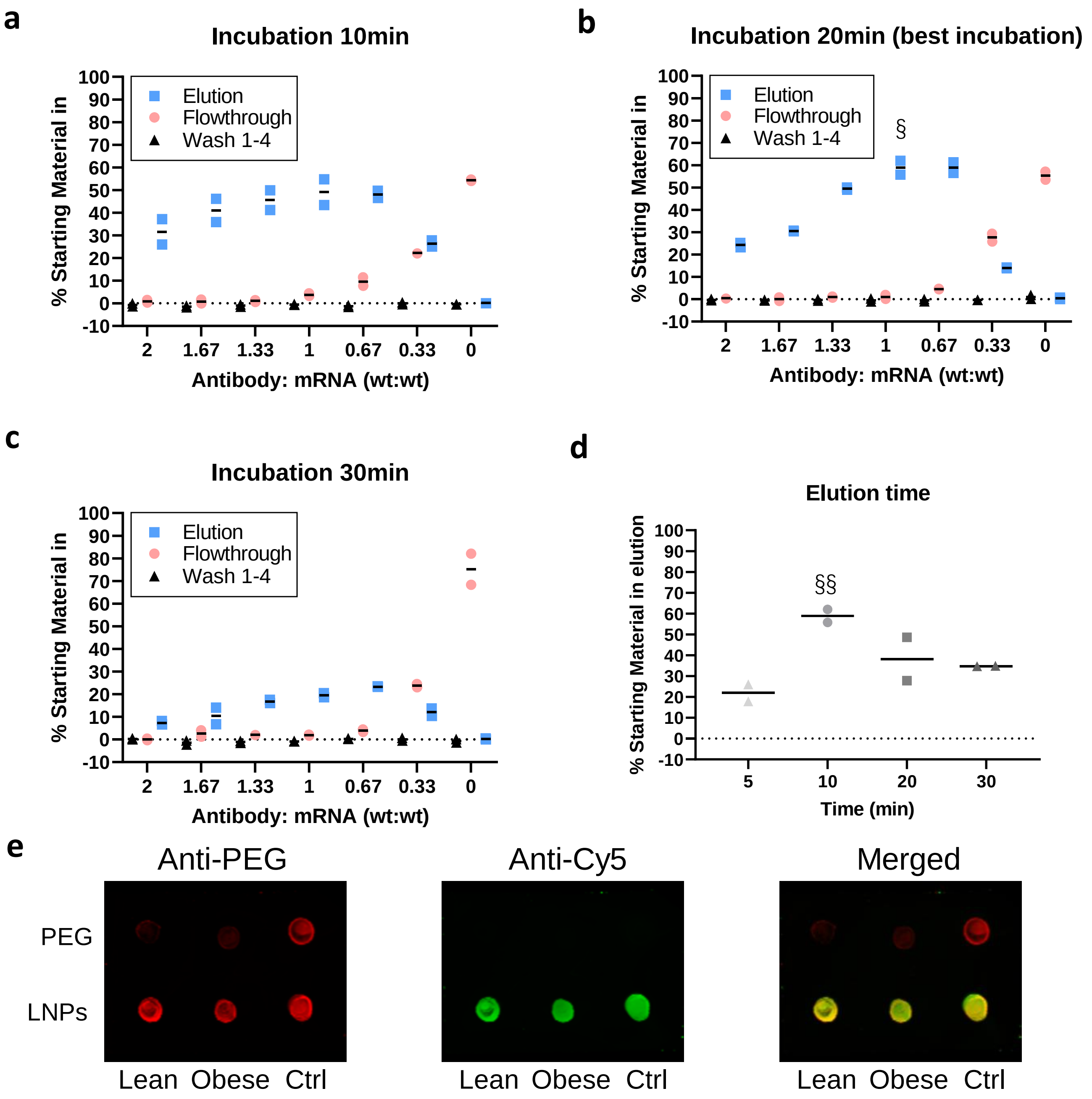

Supplementary Fig. 10. The optimization of LNPcor entity capture and elution.
a, b,c, To capture the LNPcor entities, they were incubated various amount of magnetic beads conjugated with anit-PEG antibodies to mRNA containing LNPs (Antibody: mRNA) for 10, 20 and 30 min at room temperature with gentle mixing. The beads were then thoroughly washed and incubated in elution buffer for 10 min. The recovery is illustrated as the percentage of starting material in flowthrough, wash buffer or final elution, measured using Cy5 labelled mRNA cargo. The dotted line indicated the recovery of LNPs from PBS (maximum recovery without potential plasma interference) at 20 min incubation. §,The highest recovery was achieved at 20 min incubation with an antibody to mRNA ratio of 1 (wt:wt). Mean value (black bar) derived from independent experimental replicates (n=2), with individual values indicated. Source data are provided as a Source Data file.
d, various elution times were then tested with the optimized capture conditions. The dotted line indicated the recovery of LNPs from PBS (maximum recovery without potential plasma interference) at 20 min incubation. §§,The highest recovery was achieved with 10min elution. The recovery rate in Fig. 2 was calculated according to recovery from PBS (maximum achievable recovery). Mean value (black bar) derived from independent experimental replicates (n=2), with individual values indicated. Source data are provided as a Source Data file.
e, PEG-lipid or LNPs at an equivalent concentration to the 200ng/well dose were spiked into lean and obese plasma. The recovery of free PEG-lipid was negligible compared to the LNP formulations.

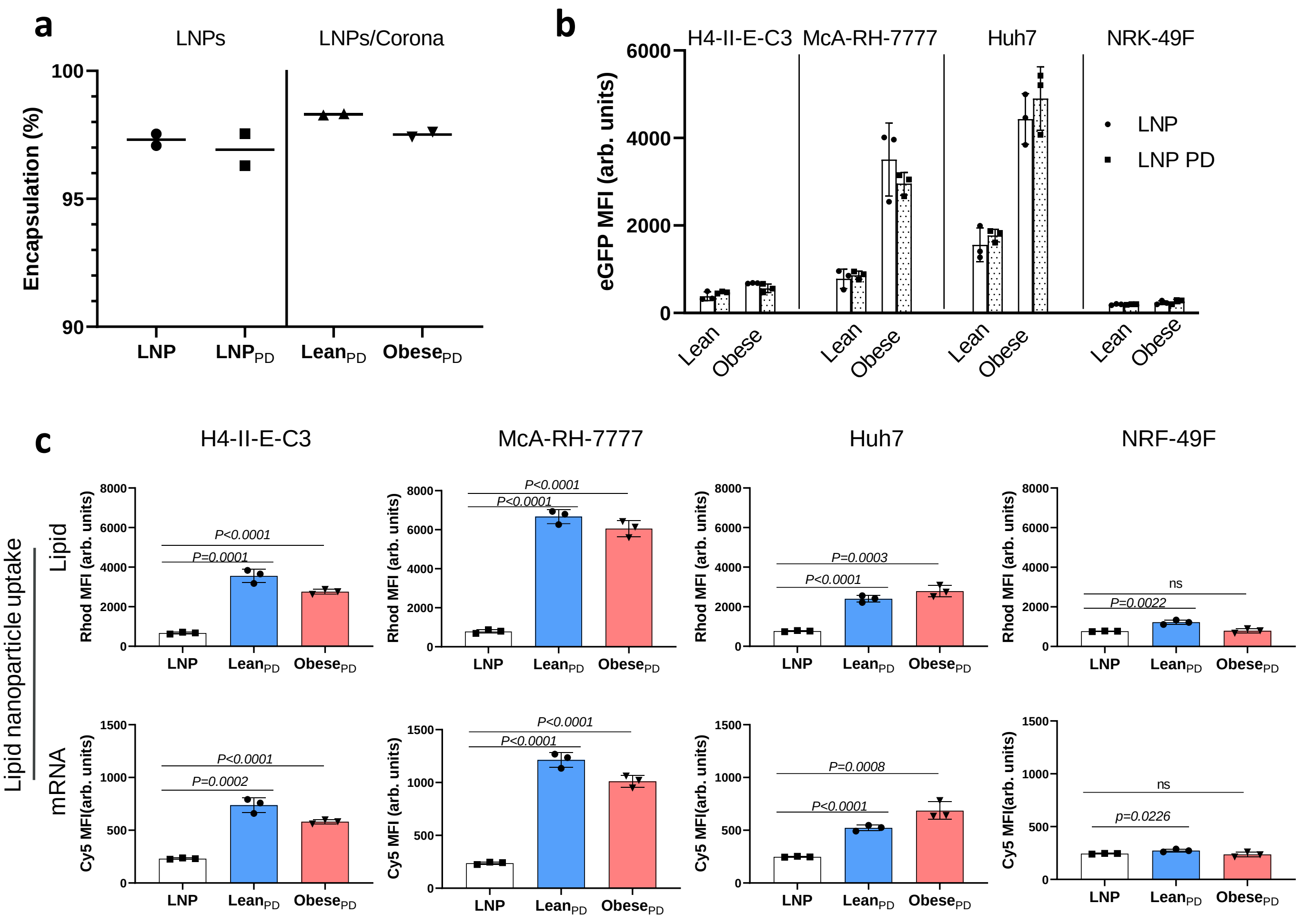

Supplementary Fig. 11. The redosing of LNPcor entities.

a, the encapsulation of mRNA by original LNPs (LNP), LNPs retrieved from PBS (LNP$_{PD}$), lean pool plasma (Lean$_{PD}$) and obese pool plasma (Obese$_{PD}$), as revealed using ultrafast isolation was similar in all cases. Mean value (black bar) derived from independent experimental replicates (n=2), with individual values indicated. Source data are provided as a Source Data file.

b, The LNP and LNP$_{PD}$ (LNP isolated from PBS without corona) were dosed to recipient cells at 200ng/well dose for 10h. The resulted eGFP expression (MFI, mean fluorescent intensity, n=3) was evaluated by high throughput imaging. The two types of LNPs provoked similar eGFP expression in recipient cells supplemented with lean pool or obese plasma without significant difference. The error bars represent standard deviation of the mean values derived from raw images (n=3 experimental replicates). Source data are provided as a Source Data file.

c, The LNPs uptake was evaluated at 200ng/well dose for 10h with or without plasma. When dosed under plasma free conditions, negligible amounts of uncomplexed original LNPs were internalized by recipient cells. In contrast, Lean$_{PD}$ and Obese$_{PD}$ resulted immediate uptake. The error bars represent standard deviation of the mean values derived from raw images (n=3 experimental replicates). Significance P values are determined by unpaired two-tailed t-test. ns: not significant. Source data are provided as a Source Data file.

a

b

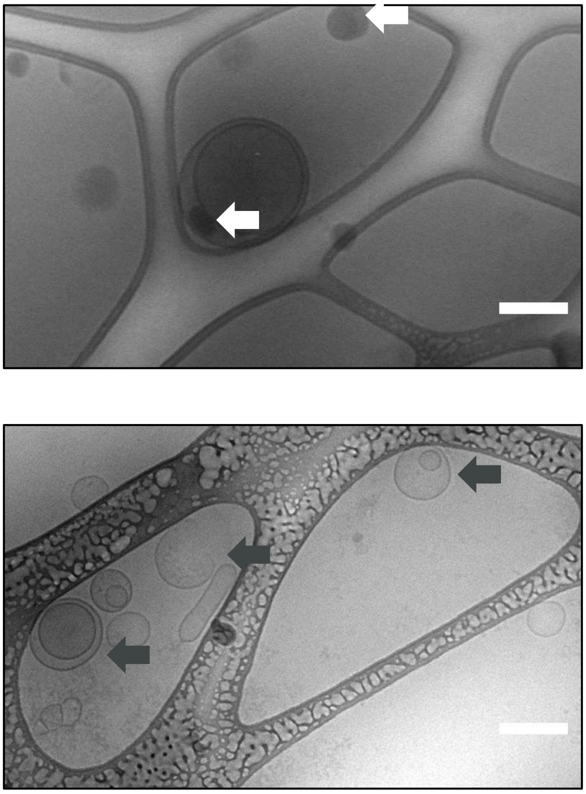

Supplementary Fig. 12. Representative Cryo-EM images of lean pool plasma derived LNPcor (a) and obese pool plasma derived LNPcor (b) selected from 20 images took from one LNPcor batch pooled from experimental replicates . Scale bar=200 nm. White arrow, Lean$_{PD}$ LNPcor complex with electron dense morphology. Dark arrow, Obese$_{PD}$ LNPcor complex with multi-laminar structures.

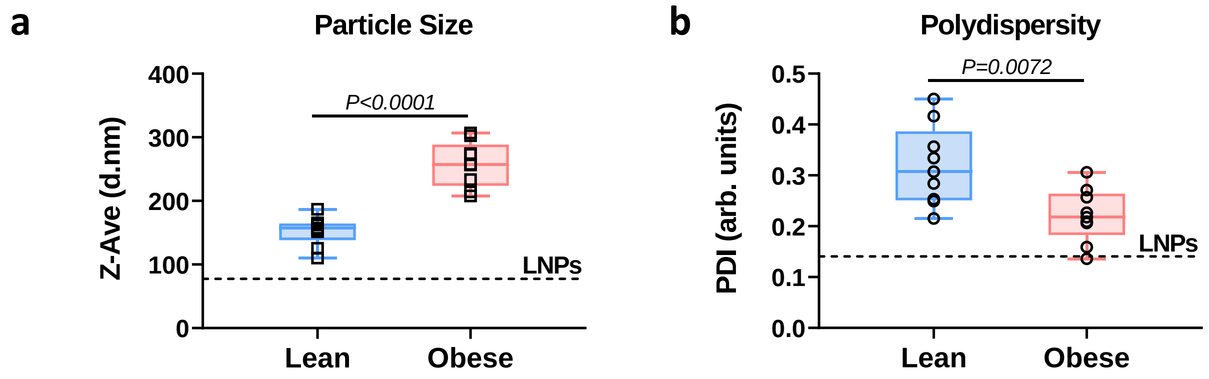

Supplementary Fig. 13. The characterization of harvested LNPcor.

a, The individual obese plasma derived LNPcor displayed larger zeta-average size than individual lean plasma derived counterparts, by DLS (n=9, 8 independent individual plasmas with 1 pooled). The box represents the interquartile range (first quartile, median and third quartile) with whiskers indicating minima to maxima. P value is obtained by two-tailed Mann-Whitney test.

b, The polydispersity of individual lean-plasma-derived LNPcor complexes was higher than individual obese-plasma-derived counterparts. The size and polydispersity of original LNPs are indicated by the dotted line (n=9, 8 independent individual plasmas with 1 pooled). The box represents the interquartile range (first quartile, median and third quartile) with whiskers indicating minima to maxima. P value is obtained by two-tailed Mann-Whitney test. Source data are provided as a Source Data file.

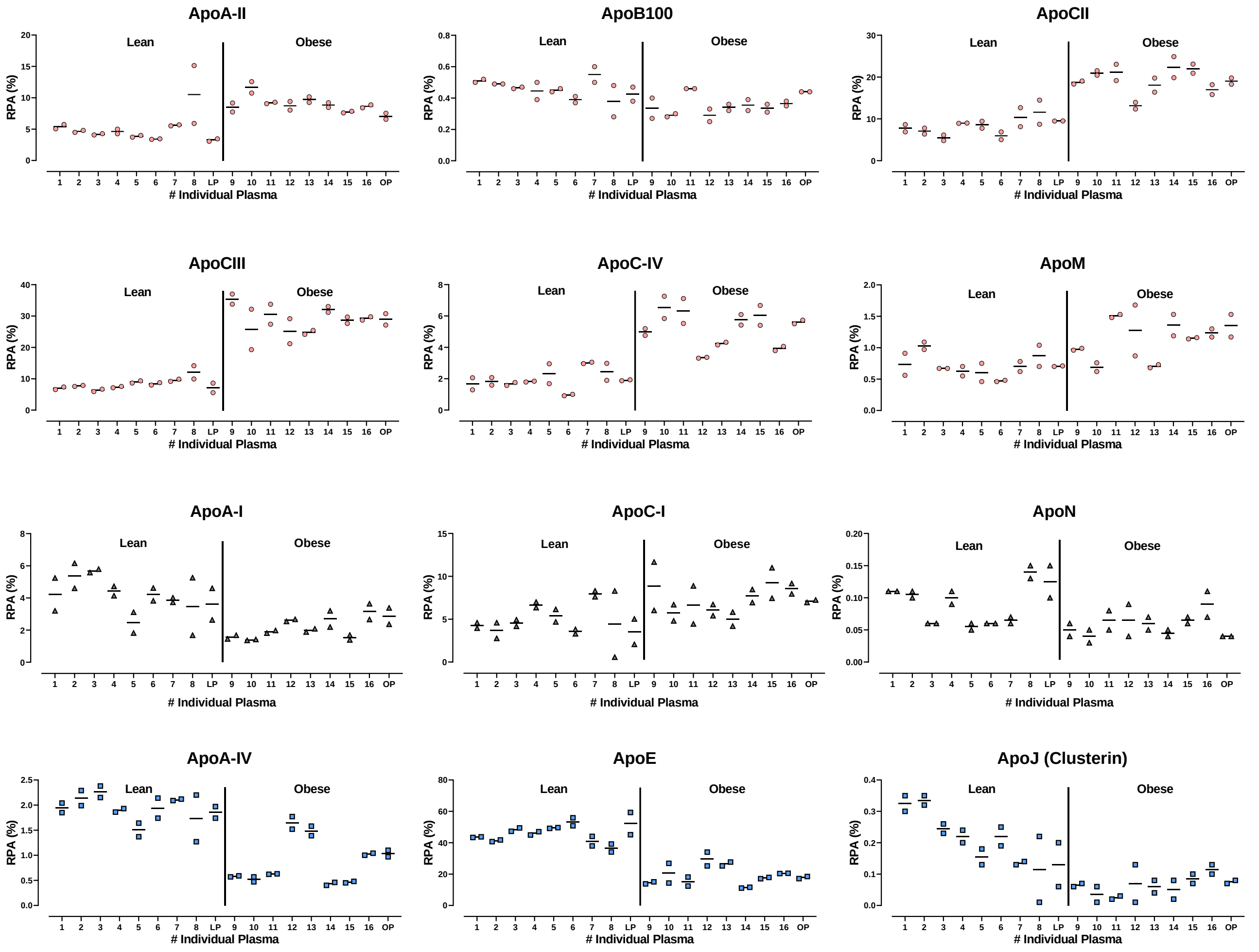

Supplementary Fig. 14. The quantification of major corona apolipoproteins of individual plasma derived LNPcor. The bar charts illustrate the RPA% of apolipoproteins identified in each individual plasma derived LNPcor. The ratio is calculated by iBAQ intensity. Mean value (black bar) derived from independent experimental replicates (n=2), with individual values indicated. Source data are provided as a Source Data file.

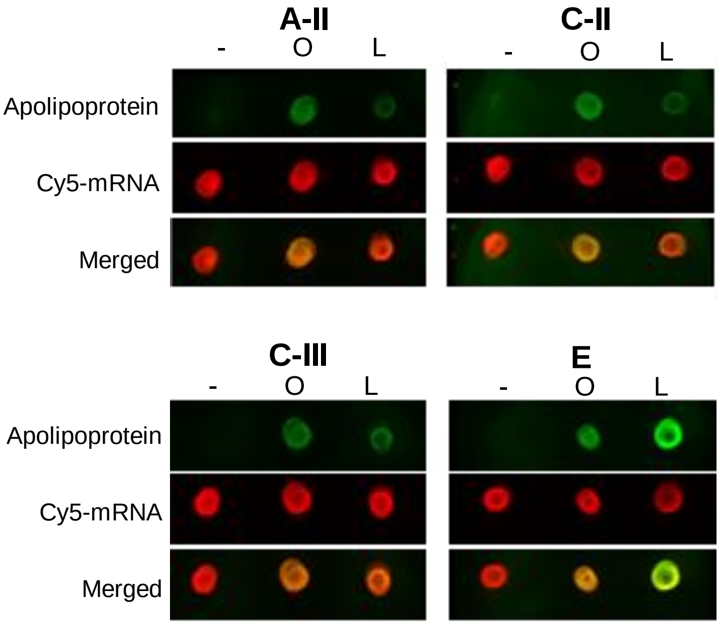

Supplementary Fig. 15. The validation of major corona proteins. Immunoblot of abundant apolipoproteins identified in LNPcor (O = obese, L = lean, - = no plasma exposure) .

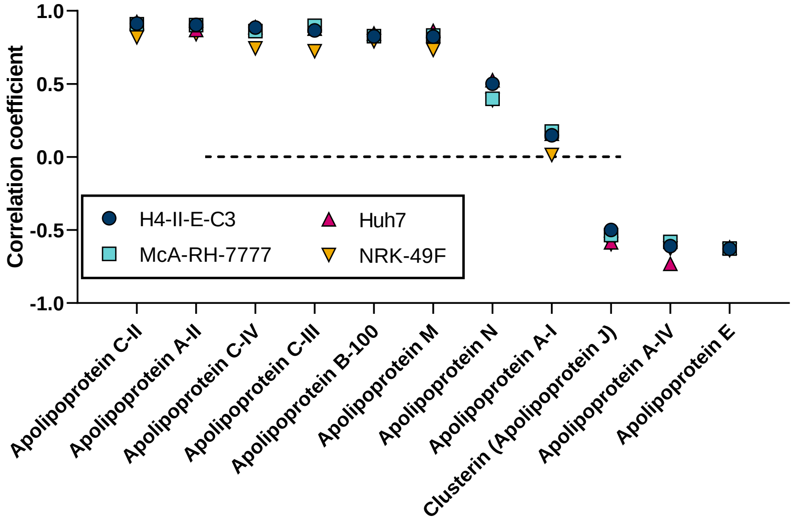

Supplementary Fig. 16. A summary of the correlation coefficient r between corona apolipoproteins in LNPcor and eGFP expression in four recipient cell lines. Source data are provided as a Source Data file.

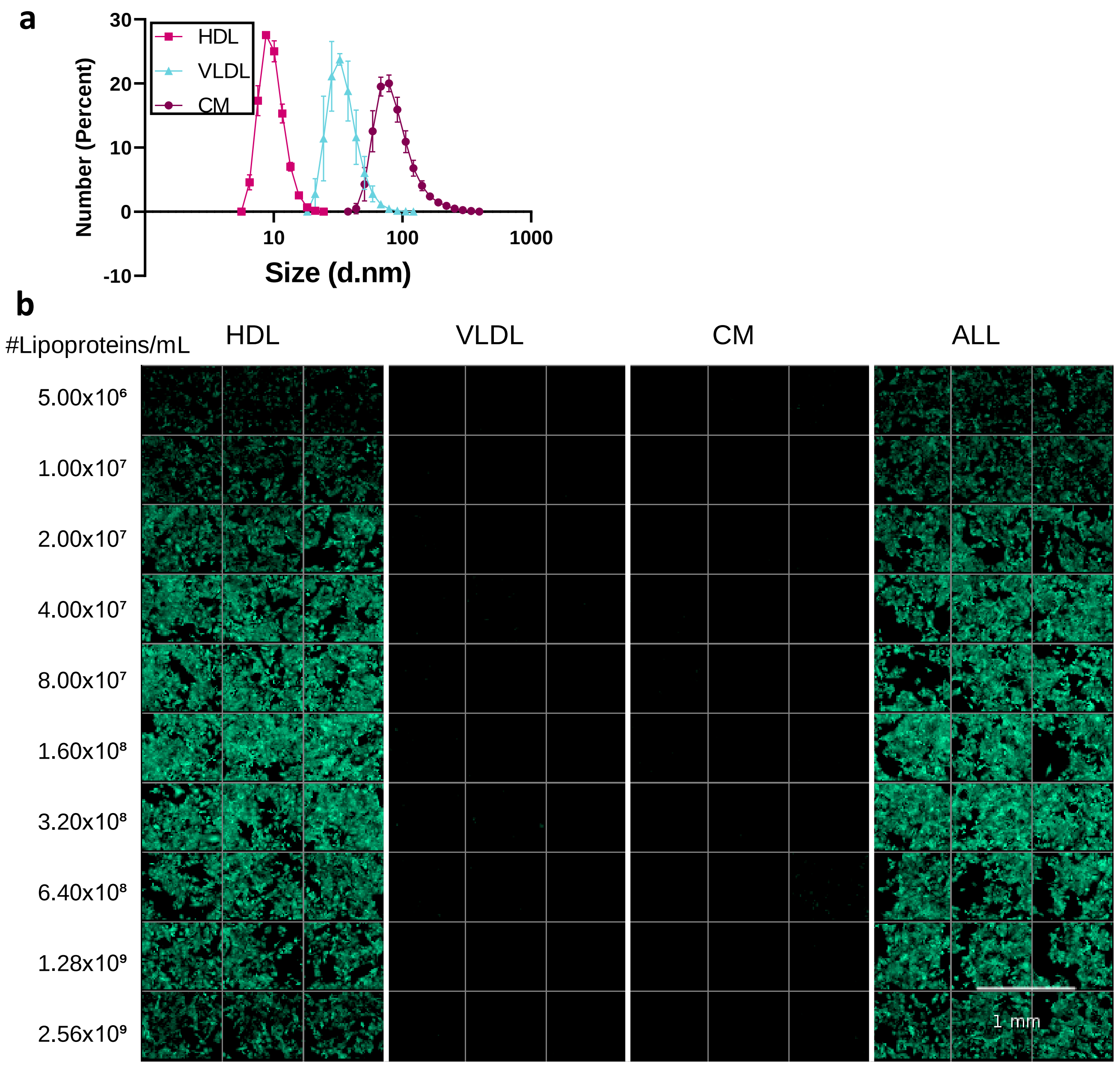

Supplementary Fig. 17. Lipoprotein spike-in.
a, the size of lipoproteins used in spike-in experiment measured using DLS. The error bars represent standard deviation of the mean (n=3 experimental replicates). Source data are provided as a Source Data file.
b, Representative images from three experiment replicates of lipoprotein spike-in introduced eGFP expression modulation.

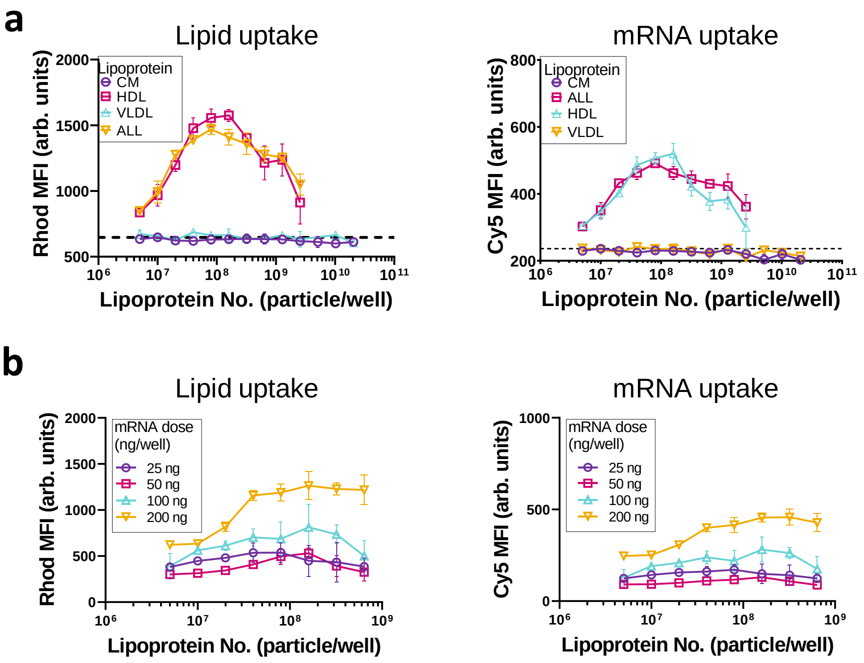

Supplementary Fig. 18. LNP uptake profile with lipoprotein spiked-in rat LP plasma.
a, The spike-in of HDL, but not VLDL and CM improved the uptake of LNPs and stimulated eGFP expression, while high HDL inhibited LNP uptake. The error bars represent standard deviation of the mean values derived from raw images (n=3 experimental). Source data are provided as a Source Data file.
b, HDL spike-in with different doses of LNPs. Lower LNP doses reached uptake plateaus at lower HDL concentration, while higher HDL:LNP ratios could inhibit LNP uptake. The error bars represent standard deviation of the mean values derived from raw images (n=3 experimental replicates). Source data are provided as a Source Data file.

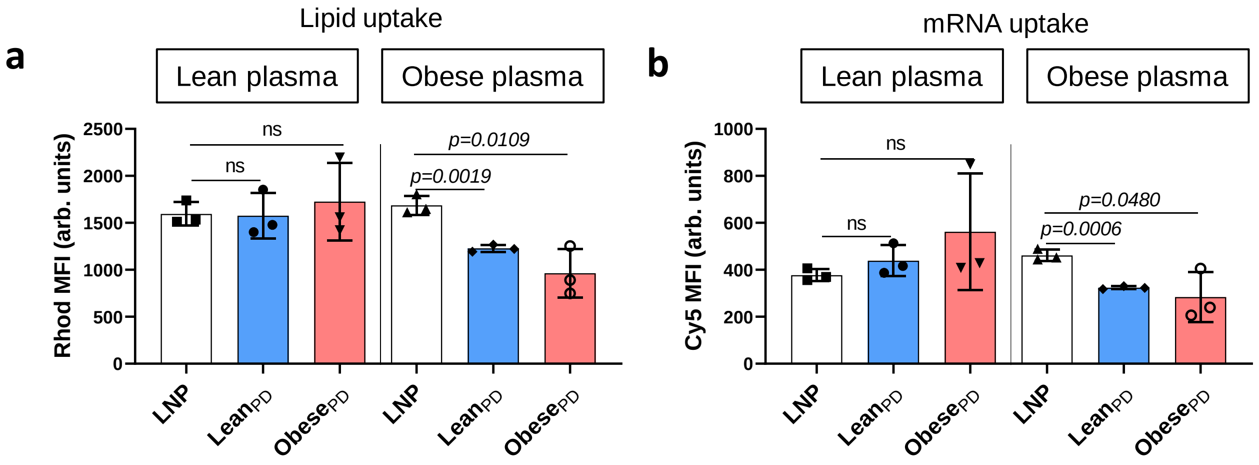

Supplementary Fig. 19. Plasma inhibited uptake of LNPcor. The lean pool plasma derived LNPcor ($Lean_{PD}$) and obese pool plasma derived LNPcor ($Obese_{PD}$) were dosed to recipient cells while supplemented with 1% lean or obese plasma respectively. The obese plasma demonstrated a clear inhibition on LNPcor internalization in terms of lipid components (**a**) and mRNA (**b**). The error bars represent standard deviation of the mean values derived from raw images (n=3 experimental replicates). Source data are provided as a Source Data file.

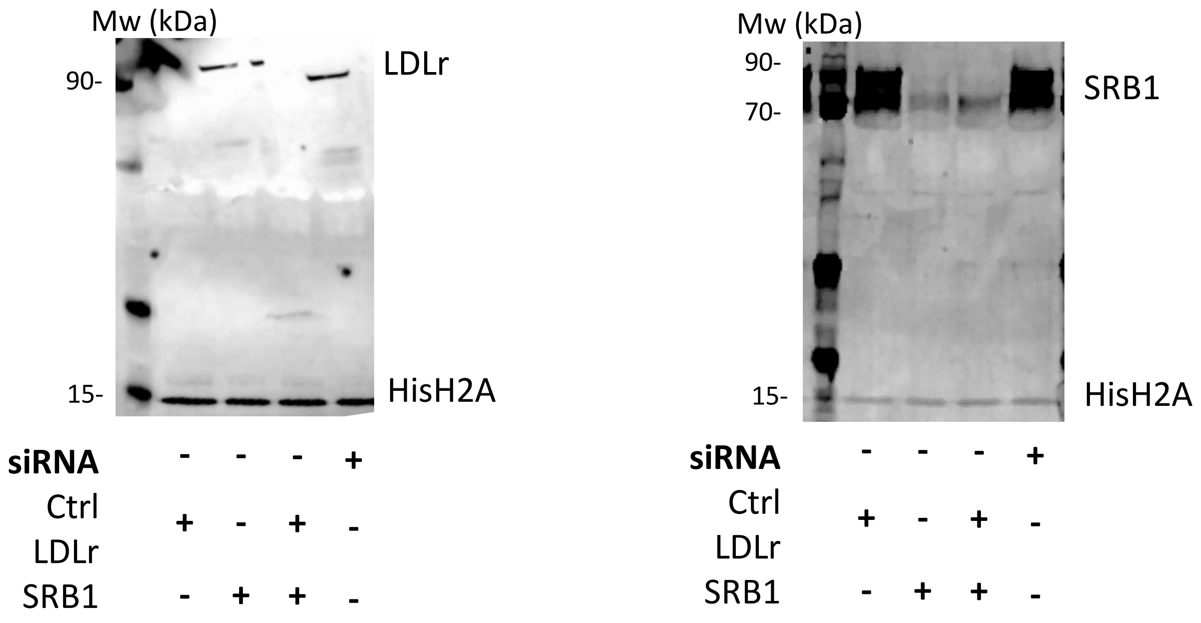

Supplementary Fig. 20. The expression level of LDLr and SRB1. The expression of LDLr and SRB1 was examined by Western blot when Huh7 hepatocytes were treated with the siRNA of interested as indicated. A scrambled sequence with no cellular target was used as the siRNA negative control. Histone H2A (HisH2A) was employed as loading control. The type of siRNA treatments are indicated as +, treated and -, untreated. The representative gel is selected from three experimental replicates. Source data are provided as a Source Data file.

Supplementary Table 2. ***In vivo*** **study design.**

Group	Treatment	Dose mg/kg	Group Size (n=)	LNP conc (mg/ml)
1	GFP	0.1	5	0.1
2	GFP/HDL	0.1	5	0.1
3	PolyA	0.1	5	0.1
