# Supplementary material for: Multiomics analysis of naturally efficacious lipid nanoparticle coronas reveals high-density lipoprotein is necessary for their function
Source: Nat Commun. 2023 Jul 6;14:4007. doi: 10.1038/s41467-023-39768-9 (PMC10325984; doi:10.1038/s41467-023-39768-9)
Supplement: Supplementary file 2 — Description of Additional Supplementary Files Document [file 41467_2023_39768_MOESM2_ESM.pdf]

Description of Additional Supplementary Files Document.

Supplementary Data 1. Rat plasma biomarker and lipid composition characterization. Obesity related biomarkers in each individual rat plasmas that used in this study were measured. The obese plasmas all demonstrated clear obese characteristics. Lipid species of each individual plasma was characterized using lipidomics as described in Methods.

Supplementary Data 2. Lipid content of individual lean and obese plasma derived LNPcor. The lipid species of each plasma derived LNPcor was characterized using lipidomics as described in Methods.

Supplementary Data 3. OPLS correlation dataset (by hits and by animals). Significantly different corona protein and lipids among all individual coronas were extracted as hits and normalized by Z-scoring. The OPLS correlation was perform between the hits and cellular eGFP expression.

Supplementary Movie 1. Nanoparticle tracking analysis movie clips of original LNPs.

Supplementary Movie 2. Nanoparticle tracking analysis movie clips of PBS (buffer for sample dilution).

Supplementary Movie 3. Nanoparticle tracking analysis movie clips of Lean LNPcor.

Supplementary Movie 4. Nanoparticle tracking analysis movie clips of lean background (non-specific pulldown from lean plasma-beads interaction).

Supplementary Movie 5. Nanoparticle tracking analysis movie clips of Obese LNPcor.

Supplementary Movie 6. Nanoparticle tracking analysis movie clips of obese background (non-specific pulldown from obese plasma-beads interaction).
