# Supplementary material for: Multiomics analysis of naturally efficacious lipid nanoparticle coronas reveals high-density lipoprotein is necessary for their function
Source: Nat Commun. 2023 Jul 6;14:4007. doi: 10.1038/s41467-023-39768-9 (PMC10325984; doi:10.1038/s41467-023-39768-9)
Supplement: Supplementary file 12 — Reporting Summary [file 41467_2023_39768_MOESM12_ESM.pdf]

nature portfolio

Corresponding author(s): Alan Sabirsh

Last updated by author(s): May 5, 2023

Reporting Summary

Nature Portfolio wishes to improve the reproducibility of the work that we publish. This form provides structure for consistency and transparency in reporting. For further information on Nature Portfolio policies, see our Editorial Policies and the Editorial Policy Checklist.

Statistics

For all statistical analyses, confirm that the following items are present in the figure legend, table legend, main text, or Methods section.

n/a	Confirmed	
☐	☒	The exact sample size (*n*) for each experimental group/condition, given as a discrete number and unit of measurement
☐	☒	A statement on whether measurements were taken from distinct samples or whether the same sample was measured repeatedly
☐	☒	The statistical test(s) used AND whether they are one- or two-sided<br>*Only common tests should be described solely by name; describe more complex techniques in the Methods section.*
☒	☐	A description of all covariates tested
☒	☐	A description of any assumptions or corrections, such as tests of normality and adjustment for multiple comparisons
☐	☒	A full description of the statistical parameters including central tendency (e.g. means) or other basic estimates (e.g. regression coefficient) AND variation (e.g. standard deviation) or associated estimates of uncertainty (e.g. confidence intervals)
☐	☒	For null hypothesis testing, the test statistic (e.g. *F*, *t*, *r*) with confidence intervals, effect sizes, degrees of freedom and *P* value noted<br>*Give P values as exact values whenever suitable.*
☒	☐	For Bayesian analysis, information on the choice of priors and Markov chain Monte Carlo settings
☒	☐	For hierarchical and complex designs, identification of the appropriate level for tests and full reporting of outcomes
☐	☒	Estimates of effect sizes (e.g. Cohen's *d*, Pearson's *r*), indicating how they were calculated

Our web collection on statistics for biologists contains articles on many of the points above.

Software and code

Policy information about availability of computer code

Data collection	Zetasizer (v7.12, Malvern), NTA (v3.0, Malvern) Cell Voyager (vR1.17.05, Yokogawa), Image Studio (v4.0, Li-Cor), MaxQuant software (v1.6.6.0, CoxLab), iTEM TIA image capture software (v4.7, Olympus)
Data analysis	Columbus Image-analysis Software (v2.9.0, Perkin-Elmer), JMP (v15.0.0, SAS), Prism (v9.0.0, Graphpad), Perseus software (v1.6.2.3, CoxLab), SIMCA (v16.0.1, Umetrics)

For manuscripts utilizing custom algorithms or software that are central to the research but not yet described in published literature, software must be made available to editors and reviewers. We strongly encourage code deposition in a community repository (e.g. GitHub). See the Nature Portfolio guidelines for submitting code & software for further information.

Data

Policy information about availability of data

All manuscripts must include a data availability statement. This statement should provide the following information, where applicable:

- Accession codes, unique identifiers, or web links for publicly available datasets
- A description of any restrictions on data availability
- For clinical datasets or third party data, please ensure that the statement adheres to our policy

The datasets generated during and/or analyzed during the current study are provided in the Source Data file and Supplementary Information. The proteomics raw files were searched against UniProt FASTA database (Rattus norvegicus UP000002494, www.uniprot.org/taxonomy/10116; Homo sapiens UP000005640,

www.uniprot.org/taxonomy/9606, where applicable). Data generated in this study have been deposited in the PRIDE Archive under accession code PXD041925 (www.ebi.ac.uk/pride/archive/projects/PXD041925), PXD041938 (www.ebi.ac.uk/pride/archive/projects/PXD041938), and PXD041944 (www.ebi.ac.uk/pride/archive/projects/PXD041944).

Human research participants

Policy information about studies involving human research participants and Sex and Gender in Research.

Reporting on sex and gender	No human research participants were included in this study
Population characteristics	No human research participants were included in this study
Recruitment	No human research participants were included in this study
Ethics oversight	No human research participants were included in this study

Note that full information on the approval of the study protocol must also be provided in the manuscript.

Field-specific reporting

Please select the one below that is the best fit for your research. If you are not sure, read the appropriate sections before making your selection.

☒ Life sciences ☐ Behavioural & social sciences ☐ Ecological, evolutionary & environmental sciences

For a reference copy of the document with all sections, see nature.com/documents/nr-reporting-summary-flat.pdf

Life sciences study design

All studies must disclose on these points even when the disclosure is negative.

Sample size	No formal sample-size calculation was performed. For imaging experiments, cell data was collected for as many as possible whilst maintaining time-points required for time-course experiments. To examine the individual differences, data acquired with individual rat plasma was kept separate.
Data exclusions	No data were excluded from the analysis.
Replication	All reported results were replicable. For imaging experiments, statistical testing was carried out upon three independent experimental replicates. For omics experiments, a minimum number of two independent experimental replicates were used for data statistics.
Randomization	No sample randomization or allocation strategy was applied
Blinding	No formal blinding was applied as all quantitative analysis was performed utilizing automated tools (Columbus, MaxQuant and Perseus software) that ensured the same criteria for imaging intensity detection, -omics quantification applied across experimental samples.

Reporting for specific materials, systems and methods

We require information from authors about some types of materials, experimental systems and methods used in many studies. Here, indicate whether each material, system or method listed is relevant to your study. If you are not sure if a list item applies to your research, read the appropriate section before selecting a response.

Materials & experimental systems

n/a	Involved in the study	
☐	☒	Antibodies
☐	☒	Eukaryotic cell lines
☒	☐	Palaeontology and archaeology
☐	☒	Animals and other organisms
☒	☐	Clinical data
☒	☐	Dual use research of concern

Methods

n/a	Involved in the study	
☒	☐	ChIP-seq
☒	☐	Flow cytometry
☒	☐	MRI-based neuroimaging

Antibodies

Antibodies used	PEG (Abcam, #ab133471, Clone PEG-2-128), Dilution 1:1000

Antibodies used	ApoAII (Abcam, #ab92478, Clone EPR2913), Dilution 1:1000<br>ApoCII (Abcam, #ab230447, Clone NA), Dilution 1:1000<br>ApoCIII (Abcam, #ab76305, Clone EP1372Y), Dilution 1:1000<br>ApoE (Abcam, #ab183597, Clone EPR19392), Dilution 1:1000<br>LDLr (Abcam, #ab30532, Clone NA), Dilution 1:2000<br>SRB1 (Abcam, #ab217318, Clone EPR20190), Dilution 1:2000<br>Histone H2A (Abcam, #ab18255, Clone NA), Dilution 1:2000<br>680RD Goat anti-Mouse IgG (LI-COR Biotechnology, #926-68070, Clone NA), Dilution 1:2000 (dot blot), 1:5000 (western blot)<br>800CW goat anti-rabbit IgG (LI-COR Biotechnology, #926-32211, Clone NA), Dilution 1:2000 (dot blot), 1:5000 (western blot)
Validation	All antibodies were validated either in the current study, by manufacturers or by previous independent publications as follows<br>PEG: Validated with PEG spike-in in manuscript (Fig. S10)<br>ApoAII: Kobayashi T et al. Glycation of HDL Polymerizes Apolipoprotein M and Attenuates Its Capacity to Bind to Sphingosine 1-Phosphate. J Atheroscler Thromb 28:730-741 (2021).<br>ApoCII: validated by the manufacturer, https://www.abcam.com/products/primary-antibodies/apolipoprotein-ciiapoc-ii-antibody-ab230447.html<br>ApoCIII:Li L et al. Proteomics analysis of potential serum biomarkers for insulin resistance in patients with polycystic ovary syndrome. Int J Mol Med 45:1409-1416 (2020).<br>ApoE: Gelibter S et al. The impact of storage on extracellular vesicles: A systematic study. J Extracell Vesicles 11:e12162 (2022).<br>LDLr: Tonini C et al. Effects of Late-Life Caloric Restriction on Age-Related Alterations in the Rat Cortex and Hippocampus. Nutrients 13:N/A (2021).<br>SRB1: Palmer MA et al. Localisation and regulation of cholesterol transporters in the human hair follicle: mapping changes across the hair cycle. Histochem Cell Biol 155:529-545 (2021).<br>Histone H2A: Pinto DO et al. Extracellular vesicles from HTLV-1 infected cells modulate target cells and viral spread. Retrovirology 18:6 (2021).<br>680RD Goat anti-Mouse IgG: David A et al. A common TMPRSS2 variant has a protective effect against severe COVID-19. Curr Res Transl Med 70:103333 (2022).<br>800CW goat anti-rabbit IgG: Snetkov X et al. A Conserved Tryptophan in the Envelope Cytoplasmic Tail Regulates HIV-1 Assembly and Spread. Viruses 14:N/A (2022).<br>More references can be found at the manufacturers' website

Eukaryotic cell lines

Policy information about cell lines and Sex and Gender in Research

Cell line source(s)	H4-II-E-C3 (CRL-1600), McA-RH-7777 (CRL-1601), and NRK-49F (CRL-1570) cells were purchased from ATCC. Huh7 (Riken - RCB1366) cells were a kind gift from Samir El-Andaloussi (Karolinska institutet, Stockholm).
Authentication	Cell lines were authenticated by STR profiling.
Mycoplasma contamination	Cell lines utilised were tested and negative for mycoplasma contamination
Commonly misidentified lines<br>(See ICLAC register)	None Used

Animals and other research organisms

Policy information about studies involving animals; ARRIVE guidelines recommended for reporting animal research, and Sex and Gender in Research

Laboratory animals	Ten week-old male lean and obese Zucker rats (Crl:ZUC-Leprfa) were purchased from Charles River Laboratories (Maryland, USA)
Wild animals	None Used
Reporting on sex	Male Zucker rats were employed in this study, while male and female Zucker rats demonstrate different metabolic signatures. In this study, for the consistency for comparison, the authors considered the difference only within male individuals. The gender of the rats is reported in the Methods
Field-collected samples	None Used
Ethics oversight	The experimental procedures were approved by the local Ethics Committee for Animal Experimentation (Gothenburg region, Sweden)

Note that full information on the approval of the study protocol must also be provided in the manuscript.
